# Perfluoroalkyl Substances (PFAS) and Lipid Metabolism in Experimental Animal Models: A Scoping Review on the Mechanisms Behind the Induced Hepatotoxicity

**DOI:** 10.3390/cimb47110944

**Published:** 2025-11-13

**Authors:** Gabriele Tancreda, Luca Campisi, Matteo Sarti, Luisa Pozzo, Andrea Vornoli

**Affiliations:** 1 Department of Health Sciences (DISSAL), University of Genoa, 16132 Genoa, Italy; 2 Department of Pharmacy, University of Pisa, 56124 Pisa, Italy; 3 Flashtox Srl, Via Tosco Romagnola 136, 56025 Pontedera, Italy; 4 Institute of Agricultural Biology and Biotechnology (IBBA), National Research Council, 56124 Pisa, Italy; andrea.vornoli@cnr.it

**Keywords:** PFAS, lipid metabolism, hepatotoxicity, oxidative stress, liver injury, liver inflammation, hepatic steatosis, legacy PFAS, emergent PFAS

## Abstract

Per and polyfluoroalkyl substances (PFAS) are a class of synthetic, persistent environmental pollutants detected in biological systems and increasingly recognized for their harmful effects on human health. The liver, being a central organ in the metabolism of xenobiotics, is profoundly affected by these compounds and is a main target of PFAS-induced toxicity. The purpose of the present Scoping Review is to investigate the multiple and complex mechanisms behind PFAS hepatotoxicity, taking into consideration evidence from preclinical in vivo models. Using electronic databases (PubMed and Google Scholar), a total of 38 studies were found eligible to be extensively explored to gather information regarding PFAS toxicity toward hepatic lipid metabolism, oxidative stress, injury and inflammation. Moreover, the parental exposure of these chemicals on the offspring will be discussed as well. As illustrated in the proposed graphical abstract, PFAS exposure has been linked to the triggering of oxidative stress phenomena, mitochondrial dysfunction and hepatic inflammatory infiltrate with sex specific effects in rodents. The predominant effects manifest as the overproduction of reactive oxygen species (ROS), the disruption of hepatic lipid metabolism, and the activation of several nuclear transcription factors involved in lipid regulation, with PPAR-α being the most prominent. Considering their strong bioaccumulative properties and persistence in both the environment and the human body, legacy and emerging PFAS should be regarded as potent toxicants with a distinctive role in the onset of metabolic diseases and as a pressing issue to be addressed within regulatory policies.

## 1. Introduction

PFAS (per- and polyfluoroalkyl substances), also known as “forever chemicals”, are a heterogeneous group of human-made compounds used in industries and consumer products since the 1940s [1].

According to the Organisation for Economic Co-operation and Development (OECD), a representative of 38 industrialized countries in America, Asia and Europe, PFAS are defined as “fluorinated substances that contain at least one fully fluorinated methyl or methylene carbon atom (without any H/Cl/Br/I atom attached to it)” (Data accessed: 22 November 2024). In their document “Reconciling Terminology of the Universe of Per- and Polyfluoroalkyl Substances: Recommendations and Practical Guidance” published in 2021, it is stated that the PFAS family counts a total of more than 4730 chemicals, comprehensive of the “legacy PFAS” perfluorooctanoic acid (PFOA) and perfluorooctanesulfonic acid (PFOS) and the “emerging PFAS”, slightly modified molecules such as the 6:2 chlorinated perfluoroalkyl ether sulfonic acid (6:2 Cl-PFESA) [2].

The high-energy C-F (carbon-fluorine) bond provides significant thermal and chemical stability to PFAS, which are likely to persist over long periods in the environment once introduced into it. Industrial wastewater treatment plants contribute directly to PFAS dissemination, contaminating soil, water, and food sources. Additionally, several industrial sectors, including food packaging, firefighting foams, water-repellent clothing, medical devices, inks and paints are considered some of the main sources of PFAS emission into the air [1,3].

Due to their high resistance, bioaccumulative and toxic properties, PFAS and their subcategories are considered a serious concern for human health [4].

Several studies have linked PFAS exposure to the onset of a wide variety of diseases and adverse health effects in humans. Relevant to the toxicity of these chemicals were found to be the magnitude, duration and individual features (i.e., sex, age, occupation) with infants and children as the most vulnerable subjects. Pathological mechanisms directly linked to PFAS exposure include the impairment of immune and endocrinological functions, the dysregulation of serum lipid profile and glucose metabolism and a higher risk of developing cardiometabolic disorders and different forms of cancer (e.g., kidney, prostate, testicles) [5].

PFAS’s pleiotropic toxicity is also relevant to the liver. This organ plays a crucial role in several vital physiological processes, including macronutrient and xenobiotic metabolism, immune system functions, and endocrinological regulation [6].

The hepatotoxicity exerted by PFAS and their subcategories is being evaluated due to the partially unknown effects of these compounds. Numerous vivo studies on rodents highlighted positive correlations between PFAS and the rise in markers of liver damage as well as an increased risk of developing steatosis and Metabolic Dysfunction-Associated Fatty Liver Disease (MAFLD) [7,8,9].

The mechanisms proposed behind the hepatotoxicity are numerous and involve the ability to bind and activate multiple nuclear receptor pathways, to promote liver enlargement, the deposition of lipid droplets in hepatocytes, the induction of oxidative stress, the dysregulation of the liver’s antioxidant enzymes and many more [9].

Taking into account PFAS broad spreading into the ecosystem and their partially undiscovered mode of action, the current review aims to identify the mechanisms behind per- and polyfluoroalkyl substances-induced hepatotoxicity in experimental animal models. The main focus has been directed on the toxicants’ ability to interfere with hepatic lipid metabolism and gene expression and elicit oxidative stress and inflammation. Furthermore, the effects of maternal PFAS exposure and the consequent metabolic alterations in the offspring will also be discussed.

## 2. Materials and Methods

The current Scoping Review was conducted following the Sigma extension for Scoping Review protocol “Preferred Reporting Items for Systematic reviews and Meta-Analyses extension for Scoping Reviews (PRISMA-ScR) Checklist” provided by the Journal “Current Issues in Molecular Biology” in the section “Instructions for Authors” [10].

Scoping reviews serve as a useful tool in scientific literature to map and provide an overview of the evidence and studies available on a specific topic. The checklist outlined in the protocol consists of 20 required items and two optional ones. Considering the pleiotropic toxicity of PFAS and the numerous contexts in which they are studied, this framework is valuable to apply.

The rationale behind exploring the hepatotoxicity of PFAS in experimental animal models through a scoping review lies in the selection of the studies. To clarify, the features of the protocol allow for a more specific investigation of a targeted organ while minimizing the interference of other exogenous factors.

### 2.1. Eligibility Criteria

**Figure 1:**
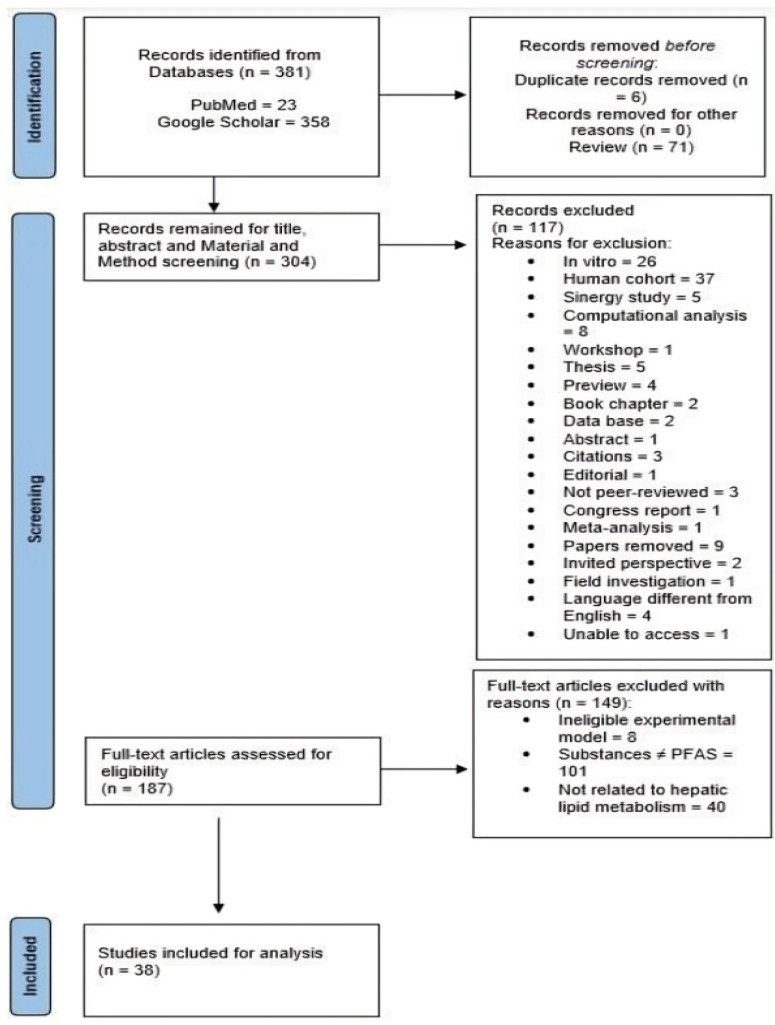
Preferred reporting items for systematic review and meta-analysis (PRISMA) flow chart of included studies. Source: Page MJ et al. BMJ 2021;372:n71. https://doi.org/10.1136/bmj.n71. The papers (*n* = 381) identified from databases PubMed and Google Scholar underwent a first rationale selection that led to the exclusion of 77 papers (*n* = 6 duplicates; *n* = 71 reviews). This led to the screening of the remaining papers (*n* = 304) for Title, Abstract and Materials and Methods Section. Coherently with the purpose of the present Scoping Review, a total of 117 studies were excluded (see the reasons for exclusion in Figure 1 above). The papers left (*n* = 187) were fully screened and assessed for eligibility based on the criteria reported in Figure 1 above. The remaining studies (*n* = 38) were confirmed as suitable to be included for the analysis and discussion.

The inclusion and exclusion criteria considered for conducting the present scoping review are provided in Figure 1. The studies’ screening process was performed by one author (G.T.) examining the title, abstract and Materials and Methods Section. Two other authors (A.V., L.P.) then revised the list of potentially eligible studies. With the intent of the review being to evaluate the hepatotoxicity of PFAS, specifically in experimental animal models, all studies whose main focus was on humans or articles that performed experiments on human cells were considered unsuitable for inclusion.

### 2.2. Search and Selection of Sources of Evidence

The screening process was conducted from the 1st of November 2024 till the 6th of January 2025 using electronic databases such as Google Scholar and PubMed. The search strategy on Google Scholar’s platform was conducted using the specific string as follows:

“(pfas OR pfos OR pfna OR pfhxs OR pfoa) AND (mouse OR rat OR in vivo OR zebrafish OR frog OR drosophila OR hamster OR rabbit OR pig) AND “hepatic lipid metabolism””. Regarding the research on the electronic database PubMed, the string used was “PFAS and hepatic lipid metabolism”, selecting 2021–2024 as the time range.

In extent, all the articles published between the years 2021 and 2024, containing simultaneously one word from the first and second bracket and the exact phrase “hepatic lipid metabolism” were considered for inclusion. Out of the 381 articles initially identified in the years of reference, only 38 were eligible to be included and analyzed in the present scoping review. The selection process was performed by examining the title, abstract and the Materials and Methods Section to assess the conformity of the studies with the purpose of our work. Mandatory criteria for the article to be included were the usage of the English language and the employment of experimental animal models to evaluate the impact of PFAS specifically on the hepatic lipid metabolism through biochemical, molecular, histological and omics techniques. All the articles included had to be related to the mechanisms of PFAS concerning the liver, its functions, steatosis, the alteration of biomarkers of oxidative stress and inflammation, and the qualitative and quantitative alterations of lipids, metabolites, proteins, and gene expression of well-established biomarkers of liver function.

### 2.3. Data Extraction and Charting

Individually, two reviewers (G.T. and, M.S.) were responsible for the data screening and extraction. Discrepancies were resolved by consensus with the other three authors (A.V., L.P. and L.C.) to ensure accuracy. Results obtained were charted according to the main pathological effects exerted by PFAS, coherently with the aims of the present work as follows:

(i) PFAS and hepatic lipid metabolism
(ii) PFAS and oxidative stress
(iii) PFAS and liver injury and inflammation
(iv) Effects of maternal PFAS exposure on the liver’s offspring

All charts concerning the studies’ examination are structured reporting:

- Year and reference of the study
- Animal model employed
- Typology of PFAS administered
- Intervention’s modality and duration
- Method of analysis
- Results

## 3. Results

### 3.1. Selection of Sources of Evidence

A total of 381 studies were identified from the two databases employed, PubMed (*n* = 23) and Google Scholar (*n* = 358), using the chosen search strings and considering the publication period from 2021 to 2024. The graphical representation of the evidence source selection process is illustrated in the PRISMA 2020 flow diagram for new systematic reviews, which includes searches of databases and registers only (Figure 1). Initially, 76 records were excluded from the analysis: 6 due to duplication and 71 because they were reviews. The remaining 304 records were screened based on their title, abstract and Materials and Methods Section. Coherently with the purpose of the present Scoping Review, 117 papers were removed (the reasons for exclusion are shown in Figure 1). In the final step of the screening process, the remaining 187 papers were screened as full-text articles: 8 of them were excluded due to the ineligibility of the experimental models, 101 studies employed substances different from PFAS and 40 were not related to the effects of PFAS on the hepatic lipid metabolism. As a result, 38 studies were considered suitable for the present Scoping Review. A summary of the included evidence and the relative data synthesis is presented in Table 1.

### 3.2. Characteristics of Sources of Evidence

Consistent with the rationale of the present study, all the included studies were conducted between 2021 and 2024 using preclinical animal models, specifically rodents (mice, *n* = 24; rats, *n* = 7), zebrafish (*n* = 7). Out of the 38 studies, 19 took place in China, 15 in the USA, 1 in Norway, 1 in the Netherlands, 1 in Luxembourg, and 1 in Finland. The chemicals administered in the different research projects belonged to the legacy and new generation’s PFAS and were given to experimental animal models singularly or in combination.

## 4. Discussion

### 4.1. PFAS and Oxidative Stress

A growing body of in vivo studies converges on a central, recurring theme: oxidative stress is a key mechanism underlying PFAS-induced toxicity, affecting diverse species and biological systems.

From zebrafish to rodents, PFAS exposure disrupts redox homeostasis, drives lipid peroxidation, and undermines antioxidant defenses, ultimately impairing liver function, metabolism, and immune regulation (Table 2). Yang et al. (2023) and Khan et al. (2023) [12,13] found that PFOS and PFOA exposure in mice activated PPARα and downstream oxidative genes like ACOX1, promoting ROS overproduction. These molecular shifts not only triggered oxidative injury but also altered lipid metabolism and liver function, highlighting the convergence between redox imbalance and metabolic dysregulation in PFAS toxicity. The findings by Gadi et al. (2023) [27] in zebrafish further support this mechanism. Low concentrations of PFOA and HFBA altered expression of stress-related genes including DDIT3, BAXA, and IL-6, implicating oxidative stress pathways such as the unfolded protein response (UPR), senescence, and inflammation. These effects were particularly pronounced in Spns1-mutant zebrafish with impaired autophagy, underlining how PFAS toxicity is exacerbated in systems with already-compromised stress responses. Chen et al. (2022) [25] extended this understanding to PFBS and PFOS, reporting severe oxidative imbalance, decreased antioxidant enzyme activity, and disrupted hepatic lipid metabolism in mice. PFOS had a more potent effect, emphasizing that both legacy and newer PFAS variants are capable of redox disruption, though to varying degrees.

Moreover, the study by Wang et al. (2022) [28] comparing PFOS and H-PFMO2OSA reaffirmed this pattern. Both compounds elevated ROS levels and impaired antioxidant enzymes like superoxide dismutase and catalase, reinforcing the notion that oxidative stress is a unifying toxicological feature across PFAS classes. Though often studied in isolation, PFAS compounds rarely exist alone in the environment. Roth et al. (2024) [22] demonstrated that exposure to a PFAS mixture significantly elevated circulating cholesterol and bile acids in mice while reducing fecal bile acid excretion. Although oxidative stress markers were not directly assessed, these metabolic disturbances, especially in conjunction with increased ASBT expression in the ileum, suggest that oxidative and inflammatory pathways may underlie impaired cholesterol catabolism and rising cardiovascular risk. Collectively, these studies paint a coherent and concerning picture: PFAS exposure consistently induces oxidative stress, a process tightly linked to lipid metabolism disruption, inflammation, and long-term organ damage. Across multiple models and exposure types, redox imbalance emerges not merely as a consequence of PFAS toxicity but as a driver of broader physiological dysfunction. Importantly, this oxidative stress does not operate in isolation; it interacts with pathways regulating bile acid cycling, immune signaling, and autophagy, potentially compounding PFAS toxicity. Given the persistence of PFAS in the environment and their bioaccumulative nature, future research should focus on the chronic and transgenerational consequences of oxidative stress induced by PFAS. There is a pressing need to identify molecular biomarkers of redox disruption and develop intervention strategies—nutritional, pharmacological, or regulatory—to mitigate the health risks posed by ongoing environmental PFAS exposure.

### 4.2. PFAS and Liver Injury and Inflammation

A robust and growing body of evidence demonstrates that exposure to both legacy and emerging PFAS induces liver injury and inflammation through diverse, yet interconnected, molecular and physiological pathways. From histopathological changes to transcriptomic and metabolomic alterations, these compounds consistently disrupt hepatic homeostasis, particularly by altering lipid metabolism, immune signaling, and gut–liver axis communication (Table 3).

He et al. (2024) [42] provided single-cell resolution of PFOS-induced hepatic changes, showing profound alterations in cell populations, especially pro-inflammatory macrophages and fibroblasts, in female mice. This immunological remodeling, paired with hepatocyte dysfunction, revealed a complex inflammatory and fibrotic response that underscores sex-specific vulnerabilities to PFAS exposure. Pan et al. (2021) and Zhao et al. (2023) [17,38] further highlighted the inflammatory potential of PFOS alternatives like 6:2 Cl-PFESA. These studies linked liver damage not only to disrupted lipid metabolism but also to gut microbiota alterations, demonstrating that PFAS can activate hepatic inflammation through microbiome-mediated endocrine and metabolic crosstalk. Similarly, Wang et al. (2022) [11] and Junaid et al. (2024) [49] confirmed that gut dysbiosis and co-contaminant exposure (e.g., nanoplastics) amplify liver injury in zebrafish models, supporting the relevance of the gut-liver axis in PFAS-induced hepatotoxicity. Attema et al. (2022) [14] and Yang et al. (2023) [12] delved into nuclear receptor signaling, showing that PFOA and GenX act through PPARα, PXR, and CAR to disrupt hepatic lipid metabolism and promote steatosis. These changes were particularly evident in high-fat diet models, suggesting that underlying metabolic stress exacerbates PFAS toxicity. This was echoed by Liu et al. (2023) [23] and He et al. (2022) [37], who demonstrated that dietary restriction or high-fat diets potentiate PFAS-induced liver injury, reinforcing the importance of nutritional status in modulating toxicity outcomes. Gadi et al. (2023) found that low, environmentally relevant levels of PFOA and HFBA upregulated inflammatory and stress-response genes such as TNF-α, IL-6, and DDIT3 in zebrafish embryos, particularly in autophagy-deficient models, suggesting that PFAS exposure may overwhelm cellular defense mechanisms and lead to early signs of MAFLD [27]. Metabolomic insights from Meng et al. (2023) [20] revealed that perinatal PFBS exposure in rats disrupts genes and metabolites related to lipid synthesis and oxidation, with potential long-term effects on liver health in offspring. Chen et al. (2022) [25] and Li et al. (2024) [35] corroborated these findings by linking PFOS and F-53B,trade neme of 6:2 Cl-PFESA and substitute for PFOS, exposure to hepatic inflammation and oxidative stress, suggesting conserved toxicity pathways across PFAS variants. Importantly, Lee et al. (2023) [15] revealed a systemic dimension to PFAS-induced hepatotoxicity: PFOS exposure not only disrupted liver metabolism but also impaired testicular function, with altered hepatokines like FGF-21 and RBP-4 linking hepatic and reproductive dysfunction. Sands et al. (2024) [40] added a novel layer by comparing the epigenetic and transcriptomic impacts of PFOA and LiTFSI, a new PFAS analog, in mice, finding that both compounds induced inflammation and lipid dysregulation, albeit through distinct molecular signatures. Finally, Roth et al. (2024) [22] demonstrated that PFAS exposure impairs bile acid cycling by downregulating intestinal ASBT, elevating circulating cholesterol and disrupting lipid clearance, which may initiate or exacerbate hepatic inflammation and metabolic liver disease.

In summary, these studies collectively paint a compelling portrait of PFAS-induced hepatotoxicity, marked by inflammation, lipid accumulation, and immune dysregulation. The convergence of evidence across species, exposure routes, and PFAS classes, legacy and novel, highlights a critical need for more comprehensive risk assessments, especially in populations facing dietary or metabolic vulnerabilities. Future research should prioritize the long-term and multigenerational consequences of PFAS exposure on liver health and identify actionable intervention points to mitigate their growing public health impact.

### 4.3. Effects of Prenatal PFAS Exposure on Offspring Health

An increasing number of studies reveal that prenatal and perinatal exposure to PFAS, whether through maternal or paternal routes, can profoundly and persistently alter the metabolic health of offspring. The findings reported in Table 4 underscore a concerning reality: early developmental windows represent critical periods of heightened vulnerability to environmental toxicants, with PFAS acting as potent disruptors of metabolic programming, immune balance, and epigenetic integrity across generations. Meng et al. (2023) [20] provided clear evidence that maternal exposure to PFBS during gestation and lactation disrupts lipid metabolism in rat offspring. Their integrative transcriptomic and metabolomic analysis revealed dysregulation in hepatic fatty acid synthesis and degradation pathways, indicating long-lasting perturbations in lipid homeostasis. These metabolic imbalances may predispose offspring to chronic metabolic conditions, highlighting the enduring impact of in utero PFAS exposure. Extending this concern beyond maternal influence, Maxwell et al. (2024) [33] demonstrated that paternal exposure to PFAS mixtures can reprogram offspring metabolism via epigenetic modifications. Specifically, altered sperm DNA methylation patterns led to transcriptional changes in liver and adipose tissue of the next generation, particularly affecting lipid metabolism and inflammatory pathways. This study not only underscores the importance of paternal environmental history but also positions sperm epigenetics as a crucial vector for transgenerational PFAS toxicity.

Fetal susceptibility is further emphasized by Blake et al. (2022) [34], who showed that gestational exposure to either PFOA or its replacement GenX impairs hepatic function in both pregnant mice and their fetuses. Fetal livers displayed distinct transcriptomic alterations, particularly in genes linked to oxidative stress, lipid metabolism, and inflammation. These data point to a direct hepatotoxic effect of PFAS during intrauterine development, with potential long-term consequences for hepatic health and systemic metabolism.

The role of the gut-liver axis in mediating developmental PFAS toxicity was explored by Liu et al. (2024) [36], who found that perinatal PFOS exposure compromised intestinal integrity and altered gut microbiota composition in offspring. These changes were linked to elevated hepatic inflammatory markers, suggesting that early-life PFOS exposure disrupts immune homeostasis via gut-liver crosstalk, a pathway increasingly recognized in chronic liver disease development.

Similarly, Yi et al. (2024) [43] identified that maternal PFOS exposure leads to hepatic lipid accumulation and inflammation in adult female offspring, driven by shifts in microbiota and impaired autophagy. These disruptions contributed to enhanced hepatic lipid deposition and chronic inflammation, reinforcing that the microbiome-gut-liver axis and autophagic processes are key mechanistic targets of developmental PFAS toxicity. Metabolic dysfunction was also evident in the findings of Shao et al. (2021) [44], where early-life PFOA exposure induced obesity and lipid dysregulation in male offspring. Remarkably, dietary supplementation with chlorogenic acid attenuated these effects by modulating metabolic and oxidative stress pathways. This not only strengthens the link between PFAS exposure and early-onset obesity but also suggests the potential for dietary interventions to mitigate PFAS-induced damage during critical developmental windows.

In summary, prenatal and early-life exposure to PFAS, whether maternal or paternal, emerges as a significant determinant of offspring health, capable of reshaping metabolic, immunological, and epigenetic landscapes. The persistent alterations observed across studies point to a heightened risk for metabolic disorders, liver inflammation, and obesity in exposed progeny. Moreover, the involvement of the gut-liver axis, autophagy, and sperm epigenetics unveils complex, multi-systemic routes by which PFAS imprint long-term biological effects. These findings call for heightened regulatory attention to reproductive PFAS exposure and further research into protective interventions that could buffer developing organisms from these enduring toxic insults.

### 4.4. Effects of PFAS on Hepatic Lipid Metabolism

The compiled findings from recent in vivo studies consistently demonstrate that both legacy and emerging PFAS compounds exert significant hepatotoxic effects across diverse species, including rodents and zebrafish, as well as various genetic and dietary models. Central to these effects is the disruption of hepatic lipid homeostasis (Table 5), mediated through a combination of direct nuclear receptor activation, notably of PPARα, and secondary consequences of oxidative stress, inflammation, and mitochondrial dysfunction. These studies also reinforce the critical roles of sex, dose, exposure duration, and host genetics in modulating susceptibility to PFAS-induced liver injury. One of the most consistent findings across studies is the elevation of serum liver enzymes—ALT and AST—as indicators of hepatocellular injury. Mouse studies by He et al. (2024) [42], Yang et al. (2023) [12], and Zhao et al. (2023) [38] all reported increased levels of these enzymes following PFOS and PFOA exposure, signaling membrane damage and cell death. Interestingly, these effects were often sex-dependent, with He et al. (2024) [42] noting greater elevations in females, whereas Khan et al. (2023) [13] found that males exhibited a greater increase in hepatosomatic index, suggesting that sex hormones or differential expression of PFAS transporters and receptors could influence toxicodynamics.

Liver enlargement, or hepatomegaly, is another common feature of PFAS exposure. Studies by Lee et al. (2023) [15], Attema et al. (2022) [14], and Kirkwood-Donelson et al. (2024) [19] documented increased liver weights across exposure to various PFAS including GenX, PFOS, and PFBA. While hepatomegaly may initially represent an adaptive response, chronic enlargement often precedes overt liver dysfunction. Disruption of bile acid metabolism—reported in Roth et al. (2024) [22] and Wang et al. (2022) [11]—adds another dimension, potentially linking hepatomegaly to impaired cholesterol turnover and enterohepatic signaling. Lipid metabolism is a particularly sensitive target of PFAS-induced hepatic toxicity. Multiple studies have demonstrated alterations in both hepatic and systemic lipid profiles, although the direction of these changes varies. Stoffels et al. (2023) [26] and Roth et al. (2024) [22] observed increased liver fat and serum cholesterol, whereas Liu et al. (2023) and Attema et al. (2024) [23,31] reported reduced plasma lipids alongside hepatic triglyceride accumulation. These discordant results likely reflect complex changes in lipid synthesis, oxidation, and transport. Indeed, transcriptomic and lipidomic analyses consistently implicate dysregulation of pathways such as fatty acid β-oxidation, lipogenesis, and cholesterol metabolism, often mediated through nuclear receptor pathways including PPARs, AMPK, and FXR. Enzymes like ACLY, ACC, and FASN frequently emerge as key nodes altered by PFAS, indicating systemic shifts in energy metabolism. Steatosis—excessive lipid deposition within liver cells—is a common histopathological outcome. Both zebrafish and rodent studies have shown PFAS-induced steatosis, suggesting that these effects are conserved across vertebrates. For instance, zebrafish exposed to PFHxS and F-53B in the studies by Liao et al. (2024) [18] and Gu et al. (2024) [30] exhibited lipid accumulation within the liver.

In rodents, He et al. (2022) [37] and Salter et al. (2021) [41] confirmed these findings with histological evidence of steatosis following PFHxS and PFOS exposure. This phenotype may reflect increased lipid synthesis, impaired fatty acid breakdown, or compromised VLDL secretion. Importantly, steatosis serves not only as a biomarker of PFAS hepatotoxicity but may also mark the early stages of non-alcoholic fatty liver disease, raising significant public health concerns.

Nuclear receptors—particularly, but not exclusively, PPARα—play a central mechanistic role in PFAS toxicity. The study by Pan et al. (2021) [17] proposes PPAR-γ and -α isoform activation as the driving factors for the promotion of hepatic metabolic lipid disorder and the consequent liver accumulation. Both transcriptional nuclear activators were found to be upregulated following 6:2 Cl-PFESA ten-week exposure period, together with genes known to play a pivotal role in triglyceride synthesis and hepatic lipid deposition. In this context, the research group highlights a marked decrease in the levels of acyl-carnitine, a central amino acid in fatty acid transport at the mitochondrial level.

Activation of PPARα promotes fatty acid oxidation and suppresses lipid synthesis and inflammation. Multiple studies have implicated PPARα activation in mediating PFAS effects. In the study by Liao et al. (2024) [18], PFHxS-induced lipid dysregulation in zebrafish was reversed by PPARα antagonism, confirming this receptor as a key molecular target. Conversely, Attema et al. (2024) [31] showed that even in PPARα-deficient mice, high-dose PFOA exposure still altered lipid profiles, suggesting that additional or parallel mechanisms may come into play depending on dose or compound. FXR and FGF15 signaling pathways, especially in bile acid regulation and hepatic glucose handling, were also implicated in studies by Wang et al. (2024) [32], indicating broader endocrine disruption.

Emerging PFAS analogs such as F-53B, GenX, and PFBA further complicate the toxicological landscape. Initially marketed as safer alternatives, many of these compounds demonstrate comparable or even enhanced hepatotoxicity. For example, F-53B exposure in studies by Wang et al. (2022) [28], Gu et al. (2024) [30], and Li et al. (2024) [35] induced steatosis, inflammation, and metabolic dysregulation, raising serious concerns about its use as a PFOS replacement. Conversely, newer compounds like LiTFSI, studied by Zhao et al. (2023) and Sands et al. (2024), showed minimal liver toxicity, indicating that not all substitutes are equally harmful [38,40]. Nonetheless, these findings highlight the need for rigorous safety evaluation of any PFAS alternative before widespread use.

Diet significantly modulates PFAS toxicity. The study by He et al. (2022) [37] demonstrates that high-fat diets can exacerbate PFAS-induced liver injury, while Deng et al. (2022) [24] showed that dietary fiber could mitigate PFOS toxicity and reduce systemic burden. Interestingly, the work by Liu et al. (2023) [23] underscores the role of chronic dietary restriction in aggravating the negative impact on liver function and parameters. These findings point to diet as a modifiable risk factor in PFAS exposure outcomes, and they emphasize the interplay between nutrition, gut microbiota, and xenobiotic metabolism. Genetic background also influences PFAS susceptibility. Use of genetically engineered models—like PPARα knockout mice or zebrafish with altered autophagy pathways—offers valuable insights into the mechanisms driving individual variation in response. Attema et al. (2022) [14] showed that PFOA exposure altered lipid metabolism even in the absence of PPARα, while Gadi et al. (2023) [27] highlighted the importance of autophagic processes in zebrafish models of PFAS exposure. These models help pinpoint key molecular pathways and may inform future risk stratification efforts. The consistency of findings across species supports a conserved mode of hepatotoxic action for PFAS. From zebrafish embryos to adult mice, PFAS exposure results in liver enlargement, enzyme elevation, lipid dysregulation, and histological damage. However, the severity and precise manifestations depend on the compound’s chain length, chemical structure, and functional group. Long-chain PFAS like PFOS and PFOA tend to bioaccumulate more readily and induce stronger effects, but short-chain and novel compounds are not necessarily benign. Their physicochemical properties influence tissue distribution, cellular uptake, and receptor binding, contributing to differential toxicity profiles.

Collectively, these findings have major regulatory and public health implications. The assumption that newer, short-chain PFAS replacements are inherently safer is increasingly being questioned. The toxicological data for GenX and F-53B, for example, suggest significant hepatic risks, warranting a more cautious approach to replacement chemistry. Given the persistence and widespread detection of PFAS in environmental and human matrices, regulatory bodies must prioritize comprehensive toxicity testing and enforce stricter exposure limits. Public health strategies should also address the role of diet and genetic predisposition in modulating risk. The study by He et al. (2024) [42] provides compelling evidence for the molecular underpinnings of PFHxS toxicity. In early life-stage zebrafish, environmentally relevant concentrations of PFHxS disrupted lipid profiles and activated the PPARα signaling pathway. Lipidomic analyses revealed dysregulation of glycerophospholipids, sphingolipids, and sterol lipids. Molecular docking studies suggested PFHxS binds PPARα with greater affinity than oleic acid, and co-exposure to a PPARα antagonist reversed lipid disruptions, strongly implicating PPARα as the molecular initiating event. Similarly, Khan et al. (2023) [13] examined chronic exposure to a PFAS mixture in A/J mice, reflecting real-world exposure conditions. Their results revealed liver accumulation of PFAS and associated increases in male body weight and hepatosomatic index. Lipidomic analysis identified 207 differentially abundant lipids, with alterations in key fatty acids. Gene and protein expression studies showed sex-specific modulation of lipid regulators, including PPARs, LXRs, RXR, FASN, and SREBP, along with altered glutathione reductase activity—pointing to both oxidative stress and disrupted nuclear receptor signaling.

In the work by Heintz et al. (2022) [29], mice were exposed to HFPO-DA (GenX) at doses up to 5 mg/kg/day. Transcriptomic analysis confirmed strong PPARα activation, with enrichment of peroxisomal and mitochondrial fatty acid oxidation genes at lower doses. Higher doses led to activation of complement, apoptosis, and cell cycle pathways, all correlating with histological liver injury. These findings confirm that GenX hepatotoxicity operates through PPARα-mediated pathways, further challenging the presumed safety of PFAS replacements.

Lastly, Hari et al. (2024) [16] explored the shared mechanisms of PFAS and PAH-induced steatosis using transcriptomic data and molecular initiating event predictions. PFAS exposure led to marked upregulation of fatty acid oxidation genes and downregulation of lipid transporters. The findings also revealed sex-specific gene responses: male rats exhibited suppression of gluconeogenesis and bile acid synthesis genes, while lipid synthesis gene Scd was upregulated. These results support a multifactorial, PPARα-centered model of PFAS-induced hepatic steatosis with clear sex-based divergence.

### 4.5. Comparison of Legacy and Emerging PFAS Hepatotoxicity Mechanisms

According to the document “Multi-Industry Per- and Polyfluoroalkyl Substances (PFAS) Study” (United States Environmental Protection Agency, 2021), emerging PFAS were introduced as alternative compounds following concerns regarding legacy PFAS’ bioaccumulative properties, environmental persistence and adverse health effects in humans [50]. Despite the efforts, these new compounds share several hepatotoxic mechanisms with the legacy ones and may pose health and environmental risks comparable to, or exceeding, those of their predecessors [51].

Our comparative analysis highlights that exposure to both long-chain PFAS, such as PFOS and PFOA, and their short-chain or alternative counterparts, including GenX, F-53B, PFBS, and PFBA, leads to the activation of PPARα [14,18,29]. As previously discussed, this nuclear receptor plays a pivotal role in regulating lipid homeostasis, and its sustained activation by PFAS results in enhanced peroxisomal fatty acid oxidation, lipid accumulation, and hepatic hypertrophy [19]. These changes are accompanied by increased oxidative stress, mitochondrial dysfunction, and depletion of antioxidant defenses, such as superoxide dismutase and catalase, culminating in hepatocellular injury and steatosis [11,35,38]. Our findings support that the fundamental hepatotoxic mode of action is conserved across PFAS classes.

However, notable distinctions emerge when comparing the capillarity of their effects. Legacy PFAS often trigger cholestatic effects and bile acid dysregulation [32]. Certain emerging PFAS, such as GenX and PFBS, appear to preferentially activate oxidative and endoplasmic reticulum stress pathways [20,27]. Moreover, newer compounds such as GenX are proposed to primarily exert their effects through PPARα activation, while the legacy compound PFOA induces hepatotoxicity via the activation of multiple nuclear receptors, including PXR and CAR [14]. Furthermore, emerging PFAS such as HFPO-DA exhibit reduced bioaccumulation compared to legacy PFAS due to their greater hydrophilicity and consequently more rapid clearance, as reported in the study by M. Thomson et al. (2019) [52].

The same result is confirmed in the study by Wang et al. (2022) [28] included in the present scoping review. After the 28-day intervention, adult male mice reported higher concentrations of PFOS compared to H-PFMO2OSA at 0.2, 1 and 5 mg/kg/day in both serum and liver. Nonetheless, emerging PFAS can elicit comparable or even stronger acute responses at equivalent molar concentrations. For instance, in the same study, the H-PFMO2OSA-treated groups suffered greater toxicological impact at the same doses than the PFOS counterparts regarding body weight, ALT and AST activity and liver histology.

Fortunately, this does not always apply to all the representatives of the emerging PFAS class. Indeed, as reported by Li et al. (2017) [53], the serum decline rate and corresponding half-life of PFHxS was remarkably longer compared to the PFOS and PFOA ones (5.3 years vs. 3.4 years and 2.7 years).

Additionally, Sands et al. (2024) [40] indicate lower levels of LiTFSI compared to PFOA in the serum of male mice after 14 and 30 days of exposure, but the same trend does not replicate for the liver compartment, where LiTFSI concentration aligns with the long-chain PFAS. Despite the shared tropism, the newer compound is associated with less profound liver toxicity compared to the legacy representative.

## 5. Conclusions

In summary, PFAS-induced hepatotoxicity is a complex, multifactorial process involving nuclear receptor signaling, oxidative stress, the activation of inflammatory pathways and metabolic reprogramming. Findings from in vivo models consistently demonstrate that both legacy and replacement PFAS can significantly disrupt liver function, often in a sex-, dose-, and diet-dependent manner. A particularly relevant aspect that emerged from delving into the PFAS-related toxicity is the transgenerational effect, supporting their view as epigenetic, immune and metabolic disruptor also in the offspring following maternal and perinatal exposure. Moreover, our findings support the notion that structural modifications do not necessarily translate into reduced hepatotoxic potency and that intra-group chemical structure variations play a distinct role in hepatotoxicity and tropism for storage in the organism. Therefore, while emerging PFAS may pose a lower risk of long-term bioaccumulation, replacing long-chain PFAS with shorter-chain or structurally modified analogs primarily reduces their persistence rather than their intrinsic toxicity, with notable exceptions. From a public health perspective, severe and proactive regulatory policies that reflect the latest mechanistic science should be applied promptly. Main goals might be represented by reducing the introduction of these compounds into the environment and promoting large-scale awareness campaigns to increase public understanding of these substances. The authors also share the opinion that while we might not be able to completely avoid the exposure of the currently present compounds in the water system and soils, the identification of novel compounds with material-wise comparable properties, while precisely characterizing the chemical groups responsible for toxicity and bioaccumulative potential, should be a key focus in the development of safer PFAS alternatives.

## Figures and Tables

**Table 1:** Summary of included studies reporting the year of publication, reference and country of the research group, paper title, animal model and PFAS administered.

Num	Year	Reference; Country	Paper Title	Animal Model (Species; Gender)	PFAS Administered
1	2022	[11]; China	Aberrant hepatic lipid metabolism associated with gut microbiota dysbiosis triggers hepatotoxicity of novel PFOS alternatives in adult zebrafish	Wild-type adult zebrafish (AB strain; ND)	PFOS; F-53B; OBS
2	2023	[12]; China	PPARα/ACOX1 as a novel target for hepatic lipid metabolism disorders induced by per- and polyfluoroalkyl substances: An integrated approach	Mice (C57BL/6J; male)	PFOS and PFOA
3	2023	[13]; Norway	Alteration of hepato-lipidomic homeostasis in A/J mice fed an environmentally relevant PFAS mixture	Mice (A/J; male and female)	PFOA; PFOS; 6:2 Cl-PFESA
4	2022	[14]; the Netherlands	Exposure to low-dose perfluorooctanoic acid promotes hepatic steatosis and disrupts the hepatic transcriptome in mice	Mice (C57BL/6J wild type and PPARα−/−; male)	PFOA; GenX
5	2023	[15]; China	PFOS-elicited metabolic perturbation in liver and fatty acid metabolites in testis of adult mice	Mice (CD-; male)	PFOA
6	2024	[16]; USA	Exposure to PFAS chemicals induces sex-dependent alterations in key rate-limiting steps of lipid metabolism in liver steatosis	Rats (Sprague Dawley; male and female)	2,3-Benzofluorene; 6:1 FTOH; PFHxSAm; 10:2 FTOH
7	2021	[17]; China	6:2 Cl-PFESA has the potential to cause liver damage and induce lipid metabolism disorders in female mice through the action of PPAR-γ	Mice (C57BL/6; female)	6:2 Cl-PFESA
8	2024	[18]; China	Perfluorohexanesulfonic Acid (PFHxS) Induces Hepatotoxicity through the PPAR Signaling Pathway in Larval Zebrafish (Danio rerio)	Zebrafish (wildtype AB strain and transgenic ApO14:GFP)	PFHxS
9	2024	[19]; USA	Investigating mouse hepatic lipidome dysregulation following exposure to emerging per- and polyfluoroalkyl substances (PFAS)	Mice (C57BL/6; male and female)	GenX; NBP2
10	2023	[20]; China	Transcriptomics integrated with metabolomics reveals perfluorobutane sulfonate (PFBS) exposure effect during pregnancy and lactation on lipid metabolism in rat offspring	Rats (Sprague Dawley; pregnant female)	PFBS
11	2024	[21]; China	Perfluorohexanesulfonic Acid (PFHxS) Impairs Lipid Homeostasis in Zebrafish Larvae through Activation of PPARα	Zebrafish (Wild-type, AB strain)	PFHxS
12	2024	[22]; USA	Exposure of Ldlr−/− Mice to a PFAS Mixture and Outcomes Related to Circulating Lipids, Bile Acid Excretion, and the Intestinal Transporter ASBT	Mice (C57Bl/6J-Ldlr−/−; male and female)	PFOA and PFOS and PFHxS and PFNA and GenX
13	2023	[23]; China	Novel insights into perfluorinated compound-induced hepatotoxicity: Chronic dietary restriction exacerbates the effects of PFBS on hepatic lipid metabolism in mice	Mice (C57BL/6; male)	PFBS
14	2022	[24]; USA	Metabolomic, Lipidomic, Transcriptomic, and Metagenomic Analyses in Mice Exposed to PFOS and Fed Soluble and Insoluble Dietary Fibers	Mice (C57BL/6J wild type; male)	PFOS
15	2022	[25]; China	Effects of perfluorobutane sulfonate and perfluorooctane sulfonate on lipid homeostasis in mouse liver	Mice (C57BL/6; male)	PFBS; PFOS
16	2023	[26]; Luxembourg	Lipidomic Profiling of PFOA-Exposed Mouse Liver by Multi-Modal Mass Spectrometry Analysis	Mice (C57BL/6NRj; male)	PFOA
17	2023	[27]; USA	Deficiency of *spns1* exacerbates per- and polyfluoroalkyl substances mediated hepatic toxicity and steatosis in zebrafish (*Danio rerio*)	Zebrafish (AB wild type and spns1-wild type (+/+), (+/−) and spns1-mutant (−/−))	PFOA, HFPA, PFTA (alone and in combination)
18	2022	[28]; China	Comparative Hepatotoxicity of a Novel Perfluoroalkyl Ether Sulfonic Acid, Nafion Byproduct 2 (H-PFMO2OSA), and Legacy Perfluorooctane Sulfonate (PFOS) in Adult Male Mice	Mice (BALB/c; male)	PFOS; H-PFMO2OSA
19	2022	[29]; USA	Evaluation of Transcriptomic Responses in Livers of Mice Exposed to the Short-Chain PFAS Compound HFPO-DA	Mice (CD1; male and female)	HFPO-DA
20	2024	[30]; China	Contribution of gut microbiota to hepatic steatosis following F-53B exposure from the perspective of glucose and fatty acid metabolism	Zebrafish (wild type, AB strain)	F-53B
21	2024	[31]; The Netherlands and Finland	Metabolic effects of nuclear receptor activation in vivo after 28-day oral exposure to three endocrine-disrupting chemicals	Mice (wild type C57BL/6J; male and female)	PFOA
22	2024	[32]; China	Perfluorooctanoic acid (PFOA) and its alternative perfluorobutanoic acid (PFBA) alter hepatic bile acid profiles via different pathways	Mice (C57BL/6N; male)	PFOA; PFBA
23	2024	[33]; USA	Mixtures of per- and polyfluoroalkyl substances (PFAS) alter sperm methylation and long-term reprogramming of offspring liver and fat transcriptome	Mice (C57BL/6; male)	PFOS and PFOA and PFNA and PFHxS and GenX
24	2022	[34]; USA	Transcriptional pathways linked to fetal and maternal hepatic dysfunction caused by gestational exposure to perfluorooctanoic acid (PFOA) or hexafluoropropylene oxide-dimer acid (HFPO-DA or GenX) in CD-1 mice	Mice (CD1; female)	PFOA; GenX
25	2024	[35]; China	Hepatotoxicity induced in rats by chronic exposure to F–53B, an emerging replacement of perfluorooctane sulfonate (PFOS)	Rats (Sprague-Dawley; male)	F-53B
26	2024	[36]; China	Perinatal exposure to perfluorooctane sulfonate and the risk of hepatic inflammation in rat offspring: Perturbation of gut-liver crosstalk	Rats (Sprague-Dawley; female)	PFOS
27	2022	[37]; China	Environmental exposure to low-dose perfluorohexanesulfonate promotes obesity and non-alcoholic fatty liver disease in mice fed a high-fat diet	Mice (C57BL/6; male)	PFHxS
28	2023	[38]; China	The toxic mechanism of 6:2 Cl-PFESA in adolescent male rats: Endocrine disorders and liver inflammation regulated by the gut microbiota-gut-testis/liver axis	Rats (Sprague-Dawley; male)	6:2 Cl-PFESA
29	2023	[39]; USA	Dose Response, Dosimetric, and Metabolic Evaluations of Replacement PFAS Perfluoro-(2,5,8-trimethyl-3,6,9-trioxadodecanoic) Acid (HFPO-TeA)	Rats (Sprague-Dawley; male and female)	HFPO-TeA
30	2024	[40]; USA	Comparative hepatotoxicity of novel lithium bis(trifluoromethanesulfonyl)imide (LiTFSI, i.e., HQ-115) and legacy Perfluorooctanoic acid (PFOA) in male mice: Insights into epigenetic mechanisms and pathway-specific responses	Mice (CD-1; male)	LiTFSI; PFOA
31	2021	[41]; USA	Perfluorooctanesulfonic Acid (PFOS) Thwarts the Beneficial Effects of Calorie Restriction and Metformin	Mice (C57BL/6; male)	PFOS
32	2024	[42]; China	Single-cell transcriptomics reveal the microenvironment landscape of perfluorooctane sulfonate-induced liver injury in female mice	Mice (C57BL/6 J; male and female)	PFOS
33	2023	[43]; China	Maternal PFOS Exposure Induces Hepatic Lipid Accumulation and Inflammation in Adult Female Offspring: Involvement of Microbiome-Gut-Liver Axis and Autophagy	Mice (CD-1; female)	PFOS
34	2021	[44]; China	Early-life perfluorooctanoic acid exposure induces obesity in male offspring and the intervention role of chlorogenic acid	Mice (ICR; female)	PFOA
35	2022	[45]; USA	Exposure to a mixture of legacy, alternative, and replacement per- and polyfluoroalkyl substances (PFAS) results in sex-dependent modulation of cholesterol metabolism and liver injury	Mice (C57BL/6J; male and female)	PFOS and PFOA and PFNA and PFHxS and GenX
36	2021	[46]; USA	An ‘Omics Approach to Unraveling the Paradoxical Effect of Diet on Perfluorooctanesulfonic Acid (PFOS) and Perfluorononanoic Acid (PFNA)-Induced Hepatic Steatosis	Mice (C57BL/6J);	PFOS; PFNA
37	2024	[47]; USA	Exploring maternal and developmental toxicity of perfluoroalkyl ether acids PFO4DA and PFO5DoA using hepatic transcriptomics and serum metabolomics	Rats (Sprague-Dawley; female)	PFO5DoA; PFO4DA
38	2024	[48]; USA	Exposure to a PFOA, PFOS and PFHxS Mixture during Gestation and Lactation Alters the Liver Proteome in Offspring of CD-1 Mice	Mice (CD-1);	PFOS and PFOA and PFHxS (alone or in combination)

**Table 2:** Schematic summary of selected studies on PFAS and oxidative stress in preclinical animal models.

Reference	Animal Model	PFAS Type	Intervention	Control	Analysis Method	Results
[13]	Mice (C57BL/6; male)	PFOA, PFOS	Male mice were randomly assigned to three groups. Treatment groups were administered PFOA or PFOS dissolved in drinking water at a dose of 1 mg/kg body weight for 35 consecutive days.	Water	Liver biochemical analysis	Increased hydrogen peroxide (H_2_O_2_) levels in the liver tissues of exposed mice.
[13]	Mice (A/J; male and female)	PFAS mixture (PFOA, PFNA, PFDA, PFuDA, PFDoDA, PFTrDA, PFTeDA, PFOS)	Mice in the exposed group were fed a standard pellet diet ad libitum six days per week, supplemented with a PFAS-exposed gel diet (3 g/mouse, once per week) for a total duration of 10 weeks.	PFAS-free gel	Liver biochemical analysis	Following PFAS exposure glutathione reductase exhibited exposure-dependent alterations in expression levels, with a significant increase in both male and female mice
[25]	Mice (C57BL/6; male)	PFOS or PFBS	Male mice were exposed to PFBS (10, 500 μg/L) or PFOS (500 μg/L) via drinking water for a total of 28 days.	Pure water	Liver biochemical analysis	The PFBS group reported a dose-dependent decrease in catalase activity. In the PFOS-exposed group, an increase in catalase activity and triglyceride levels was observed.
[28]	Mice (BALB/c; male)	H-PFMO2OSA or PFOS	Mice were exposed by oral gavage once daily for 28 days to H-PFMO_2_OSA or PFOS at 0, 0.2, 1, or 5 mg/kg/day (7 groups total)	Milli-Q water	Liver biochemical analysis	In the 1 and 5 mg/kg/d H-PFMO2OSA groups and in the 5 mg/kg/d PFOS group: reduced glutathione content and glutathione reductase activity were increased in the liver.
					Liver proteomic	The groups exposed with 1 mg/kg/day of H-PFMO2OSA and PFOS were selected for proteomic analysis. Respectively, 413 and 304 proteins were found to be differently expressed. Proteins involved in oxidation-reduction processes, lipid metabolism and glutathione metabolism were found to be the most affected in both groups.
[27]	Zebrafish (Wild type, AB strain, spns1-wt (+/+), spns1(+/−), spns1(−/−))	PFOA, HFBA and PFTA	Zebrafish embryos and spns1 mutant zebrafish embryo siblings spns1-wt (+/+), (+/−) and spns1 homozygous mutant spns1-mutant (−/−), 24 h post-fertilization, were exposed to PFAS (50–150 nM) such as PFOA, HFBA and PFTA for 48 h as indicated. PFOA or HFBA was directly added into the egg water (50 nM and 100 nM) or a combination containing PFOA, HFBA, and PFTA (50 nM each).	DMSO or Ethanol at maximum levels of treatments (*v*/*v*)	Liver gene expression	PFOA exposure alone and combined exposure to PFOA, HFBA, and PFTA impact oxidative stress in mice by modulating biomarkers related to autophagy flux and senescence.

**Table 3:** Schematic summary of selected studies on PFAS liver injury and inflammation in preclinical animal models.

Reference	Animal Model	PFAS Type	Intervention	Control	Analysis Method	Results
[42]	Mice (C57BL/6; male and female)	PFOS	Male and female mice were divided in groups and administered with different doses of PFOS for 4 weeks (1 mg/kg/day, 5 mg/kg/day, 10 mg/kg/day)	Ultrapure water	Liver histopathology	PFOS-exposure in male and female mice led to hepatocellular edema and degeneration, with binucleated cells exhibiting eosinophilic cytoplasm, nuclear fragmentation, and associated inflammatory cell infiltration.
					Serum biochemical analysis	In PFOS-treated mice: higher levels of serum amino transferases, including ALT and AST, were detected. At the dose of 10 mg/kg, PFOS induced: significant increase in AST and ALT levels in female mice compared to male mice, suggesting stronger hepatotoxicity under high-dose exposure. At lower doses (1 and 5 mg/kg), no sex differences were observed.
					Liver transcriptomic	PFOS exposure determined gene expression changes with downregulation of 156 genes and upregulation 240 genes. The upregulated genes are metabolism-related while the downregulated ones are immune system-related PFOS-treated groups showed a significant increase in marker genes of hepatic stellar cells compared to the control.
[11]	Zebrafish (Wild type, AB strain)	PFOS, F-53B, OBS	Adult zebrafish were exposed to 1 µM PFOS, F-53B, or OBS for 21 days, with fish equally distributed into two 20 L tanks (n = 2) containing dechlorinated water with 0.1% (*v*/*v*) DMSO.	Dechlorinated water with 0.1% (*v*/*v*) DMSO	Liver histopathology	PFOS and its alternatives (F-53B, OBS) induced histopathological alterations in the liver proportional to their hepatic accumulation, with F-53B causing the most severe effects.
[12]	Mice (C57BL/6; male)	PFOA, PFOS	Male mice were randomly assigned to three groups. Treatment groups were administered PFOA or PFOS dissolved in drinking water at a dose of 1 mg/kg body weight (BW) for 35 consecutive days.	Water	Liver biochemical analysis	Increased in liver injury markers: ALT, AST, ALP, GGT.
					Liver histopathology	Severe mitochondrial damage, including vacuolization, mitochondrial edema, and loss of cristae.
[17]	Mice (C57BL/6; female)	6:2 Cl-PFESA	Animals had free access to food and water and were randomly assigned to four groups receiving deionized water with 0, 1, 3, or 10 μg/L 6:2 Cl-PFESA for 10 weeks.	Deionized water	Serum biochemical analysis	6:2 Cl-PFESA induced hepatic cytoplasmic vacuolation
					Liver histopathology	6:2 Cl-PFESA preferentially bioaccumulated in the liver
[20]	Rats (Sprague-Dawley rats; male and female)	PFBS	Pregnant rats were orally administered daily doses of 5.0 and 50 mg/kg PFBS (diluted with 3% starch gel) from gestational day 1 to postnatal day 21. On postnatal day 21, all the pups were weaned and provided with unrestricted access to a standard diet. Pups were sacrificed on PND 30.	3% starch gel	Liver biochemical analysis	The 50 mg/kg PFBS treatment group exhibited mild chronic inflammation in the interlobular vessels, characterized by lymphocyte infiltration.
[22]	Mice (Ldlr−/−; male and female)	PFAS Mixture: PFOA, PFOS, PFHxS, PFNA, GenX	Male and female Ldlr mice were fed an atherogenic diet for 1 week prior to the beginning of PFAS exposure and then continued on the atherogenic diet for the remainder of the study. PFAS mixture exposure lasted for 7 weeks via their drinking water. Each of the five PFAS was present in the mixture water at a concentration of 2 mg/L and the mice were allowed to drink the water ad libitum.	Control water	Liver histopathology	Levels in plasma of TNF-α showed a significant PFAS:sex interaction: lower levels in female PFAS-exposed mice and higher levels in male PFAS-exposed mice, compared with relative control groups. Levels of MCP-1 showed a significant PFAS:sex interaction: lower levels in female PFAS-exposed mice and higher levels in male PFAS-exposed mice, compared with relative controls. Levels IL-6 showed a PFAS:sex interaction: higher levels in female PFAS-exposed mice.
[23]	Mice (C57BL/6; male)	PFBS	Mice were exposed to 50 μg/L PFBS via drinking water for 42 days (6 weeks). Animals were randomly assigned to four experimental groups: (1) water + normal diet (control), (2) water + normal diet + 50 μg/L PFBS (PFBS group), (3) water + 60% normal diet (RD group), and (4) water + 60% normal diet + 50 μg/L PFBS (RD-PFBS group)	Water + normal diet	Plasma biochemical analysis	After PFBS exposure: GOT and GPT levels exhibited a significant increase. In the RD-PFBS group: the hepatic expression of GPT showed an increase compared to the PFBS treated group. In both the PFBS and RD-PFBS groups: total bile acid (TBA) levels were significantly elevated; the RD-PFBS group showing a further increase compared to the PFBS group.
[35]	Rats (Sprague-Dawley; male and female)	F–53 B	F–53 B was prepared as a 2.5 mg/mL stock solution in DMSO and diluted with deionized water to create a gradient of concentrations (1, 10, 100, and 1000 μg/L). Each treatment group was administered with each concentration of F-53B via drinking water for 6 months.	0.004% DMSO in deionized water	Serum biochemical analysis	In rats exposed to 1000 μg/L F–53 B: hyperplasia of fibrous liver tissue and visible lymphocytes in the portal area
					Liver histopathology	Significant increase in the levels of IL-2 at doses of 100 and 1000 μg/L F-53B compared to the control. Significant increase in the levels of IL-6 in all F-53B exposed groups compared to the control. Significant increase in the levels of IL-8 at doses of 10 and 1000 μg/L F-53B compared to the control. No significant changes in the levels of IL-1β or TNF-⍺ in treated groups compared to the control
[37]	Mice (C57BL/6; male)	PFHxS	Mice were fed a high-fat diet and exposed to PFHxS via drinking water at doses ranging from 60 to 110 μg/kg of body weight for 12 weeks	Milli-Q water	Liver biochemical analysis	In the PFHxS-exposed group: more distinct signs of inflammatory infilatration and greter number of hepatic fibrotic lesions compared to the control group.
					Liver histopathology	In the PFHxS-exposed group: higher expression of pro-inflammatory cytokine IL-1β pro-and pro-fibrogenic factor Col1α compared to the control.
[38]	Rats (Sprague-Dawley; male)	6:2 Cl-PFESA	Mice were administered 50 μg/kg body weight/day 6:2 Cl-PFESA for 28 days via intragastric infusion	Milli-Q water	Liver gene expression	In the 6:2 Cl-PFESA-exposed group: inflammatory cell infiltration
[40]	Mice (CD-1; male)	LiTFSI; PFOA	Mice were exposed for 14 days to 10 or 20 mg/kg body weight of LiTFSI or PFOA or for 30 days to 1 or 5 mg/kg body of LiTFSI and PFOA.	Corn oil	Liver histopathology	In PFOA-exposed 14 and 30 days groups: inflammatory infiltration, necrosis, hemorrhage, cytoplasmic vacuolation, central vein congestion, and fatty degeneration. In the LiTFSI-exposed 14 and 30 days groups: central vein dilation and necrosis (only in the 30 days group).
[15]	Mice (CD-1; male)	PFOS	During the 21-day experiment period, treated mice were administered with either 0.3 or 3 mg/g body weight of PFOS by oral gavage.	Corn oil	Liver histopathology	PFOS-exposed groups reported significant increases in polyunsaturated fatty acids, including eicosa-5,8,11-trienoic acid, eicosa-5,11,14-trienoic acid (arachidonic acid), dihomo-α-linolenic acid, and dihomo-γ-linolenic acid, along with elevated levels of oxidized ceramide, diacylglycerol, and phospholipids (phosphatidylcholine and phosphatidylethanolamine).
[27]	Zebrafish (Wild type, AB strain, spns1-wt (+/+), spns1(+/−), spns1(−/−))	PFOA, HFBA and PTFA	Zebrafish embryos and spns1 mutant zebrafish embryo siblings spns1-wt (+/+), (+/−) and spns1 homozygous mutant spns1-mutant (−/−), 24 h post-fertilization (hpf) (n = 10–16), were exposed to PFAS (50–150 nM) such as PFOA, HFBA and PFTA for 48 h as indicated. PFOA or HFBA was directly added into the egg water (50 nM and 100 nM) or a combination containing PFOA, HFBA, and PFTA (50 nM each).	DMSO or Ethanol at maximum levels of treatments (*v*/*v*)	Liver histopathology	PFOA exposure in AB-wt zebrafish embryos did not induce the gene expression of pro-inflammatory biomarkers. Differently, in spns1-wt (+/+) and (+/−), PFOA exposure determined the upregulation of edem1 and TNFα. The greater impact was reported in spns1-mutant (−/−) zebrafish embryos. HFBA-exposed AB-wt embryos reported an important upregulation of IL-6 expression. The same compound was found responsible for the induction of TNFα, IL-1β and IL-6 with a in Spns1-wt (+/+) and (+/−), with an even greater impact in Spns1-mutant (−/−) zebrafish embryos.
[14]	Mice (C57BL/6J wildtype and PPARα(−/−); male)	PFOA and GenX	Mice were given 0.05 or 0.3 mg/kg body weight/day PFOA, or 0.3 mg/kg body weight/day GenX while being fed a high-fat diet for 20 weeks	Drinking water	Serum biochemical analysis	High-dose PFOA-exposed mice presented a significant upregulation of PPARα target genes Cd36, Cyp4a14, Ehhadh, and Lpl. Low-dose PFOA-exposed mice presented Cyp4a14 increased expression. GenX-treated mice showed increased levels of Cd36, Cyp4a14, Ehhadh, and Lpl mRNA and endocrine factor Fgf21 only in wild-type animals. Transcriptomic analysis revealed that PFOA and GenX effects on hepatic gene expression are mostly or completely mediated by PPARα. PFOA and GenX exposure also regulated the hepatic expression of SREBP-1 target genes involved in fatty acid synthesis.
[25]	Mice (C57BL/6; male)	PFOS or PFBS	Male mice were exposed to PFBS (10, 500 μg/L) or PFOS (500 μg/L) via drinking water for a total of 28 days.	Pure water	Serum biochemical analysis	The PFBS (500 μg/L) exposed group showed increased apoptosis, while the treatment with PFOS (500 μg/L) induced lipid droplet accumulation, inflammation, and apoptosis.
					Testis lipidomic	

**Table 4:** Schematic summary of selected studies on prenatal PFAS exposure on offspring health.

Reference	Animal Model	PFAS Type	Intervention	Control	Analysis Method	Results
[20]	Rats (Sprague-Dawley rats; male and female)	Perfluorobutane sulfonate (PFBS)	Pregnant rats were orally administered daily doses of 5.0 and 50 mg/kg PFBS (diluted with 3% starch gel) from gestational day 1 to postnatal day 21. On post natal day 21, all the pups were weaned and provided with unrestricted access to a standard diet. Pups were sacrificed on post natal day 30.	3% starch gel	Liver histopathology	The 50 mg/kg PFBS treatment group exhibited mild chronic inflammation in the interlobular vessels, characterized by lymphocyte infiltration.
[33]	Mice (C57BL/6; male)	PFAS mixture: PFOS, PFOA, PFNA, PFHxS and GenX	The parental male mice were put on a high-fat diet (ad libitum) one week prior and during the administration of a mixture of 5 PFAS (PFOS, PFOA, PFNA, PFHxS and GenX) at a concentration of 20 µg/L each via drinking water	Water alone	Liver transcriptomic	In male offspring of PFAS-treated parental mice: identification of 40 differentially expressed genes involved in cholesterol and xenobiotic metabolism, biosynthesis of small molecule and mitotic cell cycle. In female offspring of PFAS-treated parental mice: identification of 9 differentially expressed genes with no significant enrichment.
					Liver biochemical assay	In male offspring of PFAS-treated parental mice: mean cholesterol levels were higher compared to the control. In female offspring of PFAS-treated parental mice: no significant differences in hepatic cholesterol concentration.
[34]	Mice (CD-1; pregnant female)	PFOA or GenX	Pregnant mice were daily oral gavage with 1 or 5 mg/kg PFOA or 2 or 10 mg/kg GenX from gestational day 1.5 to gestational day 17.5.	Deionized water	Liver transcriptomic	Number of significant differentially expressed genes in maternal livers: 8 at 1 mg/kg PFOA, 341 at 5 mg/kg PFOA, 93 at 2 mg/kg GenX, 253 at 10 mg/kg GenX. Number of significant differentially expressed genes in fetal livers: 69 at 1 mg/kg PFOA, 129 at 5 mg/kg PFOA, 154 at 2 mg/kg GenX, 170 at 10 mg/kg GenX. Furthermore, a total of 30 genes related to metabolism, peroxidation, synthesis, transport, and regulation of fatty acids and lipids were identified to be significantly differentially expressed in both maternal and fetal livers at all doses except 1 mg/kg PFOA.
[36]	Rats (Sprague-Dawley; pregnant female)	PFOS potassium solution prepared in 3% starch	PFOS potassium solution prepared in 3% gel starch (0.3 mg/kg) was administered daily to dams via oral gavage from gestational day 1 to post-natal day 21. On post-natal day 28 pups were sacrificed.	3% starch gel	Body and liver weight	Pups of the PFOS-exposed group showed a decreased trend in birth body weight and an increased liver index compared to the control (no statistical significances).
					Serum biochemical analysis	In the PFOS-exposed group’s offspring: significant increase in the AST levels compared to the control. No significant differences among other biomarkers (TG, LDL, HDL, ALT, ALT/AST) nor sex-dependent differences.
					Liver histopathology	In PFOS-exposed offspring: presence of inflammatory cells infiltration compared to the control.
					Serum metabolomic analysis	In PFOS-exposed offspring: 48 metabolites were significantly altered compared to the control group (31 down-regulated and 17 up-regulated). Key metabolite changes: increase in (−)-Jasmonic acid and decrement of L-leucine, L-valine, Linatine, Methyl jasmonate.
					Liver metabolomic analysis	In PFOS-exposed offspring: 62 differentially expressed metabolites compared to the control (28 down-regulated and 34 up-regulated). Key metabolites changes: CMP-N-trimethyl-2-aminoethylphosphonate and decrement of glutathione disulfide, oxidized glutathione, phosphonoacetate.
					Liver transcriptomic	In PFOS-exposed offspring: 289 differentially expressed genes compared to the control (124 up-regulated and 165 down-regulated). The most enriched biological process regards the immune response.
[43]	Mice (CD-1; pregnant female)	PFOS	Pregnant mice were administered either with a low dose 0.3 mg/kg/day or a high dose 3 mg/kg/day of PFOS from gestational day 11 to 18 via oral gavaged drinking water. Female offspring were fed a normal diet and then sacrificed at post natal day 98	Milli-Q Water	PFAS concentration	Intergenerational PFOS-exposed offspring: significantly higher concentration of the xenobiotic compared to the control group.
					Body and liver weight	No significant changes in the intergeneration PFOS-exposed offspring and control group.
					Serum biochemical analysis	Intergenerational PFOS-exposed offspring: significantly higher concentrations of ALT, AST and triglycerides levels compared to the control.
					Liver biochemical analysis	Intergenerational PFOS-exposed offspring: increased levels of total cholesterol and reduced low density lipoprotein compared to the control group.
					Liver histopathology	Intergenerational PFOS-exposed offspring: altered hepatocytes and inflammatory cell infiltration. Is also noted an increase in F4/80 cells. Furthermore, oil-red O staining confirmed higher lipid droplets accumulation compared to the control group.
					Liver gene expression	Intergenerational PFOS-exposed group: upregulation of pro-inflammatory factors. In the 0.3 mg/kg/day group: significant increase in the levels of TNF-α compared to both the control group and high dose treatment group. In the 3 mg/kg/day group: significant increase in the levels of IL-1β compared to both the control and low dose group.
					Liver protein and gene expression	PFOS treatment induced higher expression of genes and relative proteins involved in fatty acids and triglycerides transport (Cd36 and FTTP), triglycerides synthesis (Gpam), fatty acids β-oxidation (PPARα, CPT1A, ACOX1) and fatty acids synthesis (LXRα, FASN and ACC) compared to the control group. At doses of 3 mg/kg/day, PFOS treatment induced also a significant increase in the expression of SREBP2 compared to the control.
					Liver protein expression	PFOS treatment induced a dysregulation in the autophagic activity with a significant downregulation of Beclin1, upregulation of p62 and of the ratio LC3 II/I and significant inhibition of AMPK pathway in favor of m-TOR compared to the control group.
[44]	Mice (ICR; female)	PFOA	Female mice were administered with 0.05 mg/kg/day PFOA (dissolved in corn oil) via oral gavage from day 13 of pregnancy till delivery. Male offspring was fed with a normal diet and then sacrificed 12 weeks after birth.	Corn oil	Corporal composition	PFOA-exposed offspring: increased fat mass and hypertrophy of white adipocytes.
					Indirect calorimetry analysis	PFOA-exposed offspring: decrease in energy expenditure during the dark cycle period compared to the control
					PFOA concentration	PFOA accumulation in the offspring was predominant in the liver followed by the spleen and adipose tissue.
					Serum biochemical analysis	PFOA-exposed offspring: increased levels of ALP, AST and ALT compared to the control group.
					Liver histopathology	PFOA-exposed offspring: increased number of lipid vacuoles and triglycerides compared to the control.
					Liver transcriptomic	Gestational PFOA exposure influenced the expression of 1416 genes (807 upregulated and 609 downregulated) compared to the control. The upregulated pathways were in regards of insulin resistance, non-alcoholic fatty liver disease, PPAR signaling pathway and lipid metabolism.
					Liver protein expression	PFOA-exposed offspring: increased levels of NLRP3 inflammasome (NLRP3, cleave-caspase-1) compared to the control group.
					Liver histopathology	Gestational PFOA exposure led to an increase in F4/80, a marker of inflammation, in the liver of male offspring.
					Liver gene expression	PFOA-exposed offspring: increase in the relative mRNA levels of inflammation-related genes (TNFα, IL-6, Cd14, TLR4)

**Table 5:** Schematic summary of selected studies on PFAS and hepatic lipid metabolism in preclinical animal models.

Reference	Animal Model	PFAS Type	Intervention	Control	Analysis Method	Results
[42]	Mice (C57BL/6; male and female)	PFOS	Male and female mice were divided in groups and administered with different doses of PFOS for 4 weeks (1 mg/kg/day, 5 mg/kg/day, 10 mg/kg/day)	Ultrapure water	Serum biochemical analysis	Higher levels of serum amino transferases—including ALT and AST—were detected in PFOS-treated mice. At the dose of 10 mg/kg, PFOS induced a significant increase in AST and ALT levels in female mice compared to male mice, suggesting stronger hepatotoxicity under high-dose exposure. At lower doses (1 and 5 mg/kg), no sex differences were observed.
					Body and liver weight	In both sexes, PFOS exposure determined a significant increase in the ratio of liver to body weight compared to the control. The effect was more pronounced in the females.
					Liver transcriptomic	PFOS exposure determined gene expression changes with downregulation of 156 genes and upregulation 240 genes. The upregulated genes are metabolism-related while the downregulated ones are immune system-related. PFOS-treated groups showed a significant increase in marker genes of hepatic stellar cells compared to the control.
[11]	Zebrafish (Wild type, AB strain)	PFOS, F-53B, OBS	Adult zebrafish were exposed to 1 µM PFOS, F-53B, or OBS for 21 days, with fish equally distributed into two 20 L tanks (n = 2) containing dechlorinated water with 0.1% (*v*/*v*) DMSO.	Dechlorinated water with 0.1% (*v*/*v*) DMSO	Liver biochemical analysis	PFOS and its alternatives (F-53B, OBS) induced alterations in liver function indices proportional to their hepatic accumulation, with F-53B exhibiting the highest bioaccumulation and toxicity. F-53B and PFOS disrupted hepatic lipid metabolism, leading to an imbalance between lipid synthesis and oxidation
					Liver transcriptomic and gene expression	Transcriptomic analysis indicated that the disruption of hepatic lipid metabolism caused by F-53B and PFOS was mediated through PPARγ activation, leading to downstream transcriptional changes and an imbalance between lipid synthesis and oxidation.
[12]	Mice (C57BL/6; male)	PFOA, PFOS	Male mice were randomly assigned to three groups. Treatment groups were administered PFOA or PFOS dissolved in drinking water at a dose of 1 mg/kg body weight (BW) for 35 consecutive days.	Water	Serum and liver biochemical analysis	Increased in serum concentrations of triglycerides, NEFA, and glucose measured within 24 h.
					Liver transcriptomic and gene expression	PFOA exposure led to perturbation in fatty acid β-oxidation pathway and to a significant increase in ACOX1 expression.
[13]	Mice (A/J; male and female)	PFAS mixture (PFOA, PFNA, PFDA, PFuDA, PFDoDA, PFTrDA, PFTeDA, PFOS)	Mice in the exposed group were fed an AIN-93G pellet diet ad libitum six days per week, supplemented with a PFAS-exposed gel diet (3 g/mouse, once per week) for a total duration of 10 weeks.	PFAS-free gel	Body and liver weight	Increase in hepatic NEFA and triglyceride levels, corrected for protein content
					Liver lipidomic	PFAS-exposed male and female groups, respectively, reported 95 and 72 differently expressed lipids compared to their control. In both sexes, the major classes of altered lipids were diradylglycerols, glycerophosphocholines, glycerophosphoethanolamines, glycerophosphoserines, sphingomyelins, sterols, and triradylglycerols.
[14]	Mice (C57BL/6J wildtype and PPARα(−/−); male)	PFOA or GenX	Mice were given 0.05 or 0.3 mg/kg body weight/day PFOA, or 0.3 mg/kg body weight/day GenX while being fed a high-fat diet for 20 weeks	Water	Body and liver weight	Exposure to high-dose PFOA decreased body weight and increased liver weight in wildtype and PPARα(−/−) mice
					Plasma biochemical analysis	High-dose but not low-dose PFOA reduced plasma triglycerides and cholesterol
					Liver biochemical analysis	PFOA and GenX increased hepatic triglycerides
[15]	Mice (CD-1; male)	PFOS	Mice were administered 0.3 and 3 μg/g body weight of PFOS for 21 days	Corn oil	Body and liver weight	A significant increase in absolute and relative liver weights was observed at the high-dose (3 mg/g), with no noticeable effect on body weight changes
					Plasma and liver biochemical analysis	High-dose PFOS exposure significantly increased hepatic triglyceride levels and elevated serum insulin levels, while fasting blood glucose remained unchanged
[17]	Mice (C57BL/6; female)	6:2 Cl-PFESA	Animals had free access to food and water and were randomly assigned to four groups receiving deionized water with 0, 1, 3, or 10 μg/L 6:2 Cl-PFESA for 10 weeks.	Deionized water	Serum biochemical analysis	Serum metabolic profiling revealed increased amino acids and decreased acyl-carnitines following 6:2 Cl-PFESA exposure
					PFAS quantification	6:2 Cl-PFESA preferentially bioaccumulated in the liver
					Liver gene expression	10 weeks 6:2 Cl-PFESA exposure led to upregulation of Fat, fatp2 and fabp1 involved in hepatic lipid metabolism and gpat and mtp related to triglycerides synthesis. All 6:2 Cl-PFESA-treated groups reported a remarkable activation of the ppar-α and fas expression. 10 μg/L 6:2 Cl-PFESA exposure group reported increased levels of cpt1α, acox and lcad expressions. The 3 and 10 μg/L 6:2 Cl-PFESA-treated groups reported increased expressions of ppar-γ and srebp1c transcriptional activators.
[19]	Mice (C57BL/6; male and female)	GenX or NBP2	Mice were orally exposed for 28 or 30 days to GenX (100 mg/kg/day), NBP2 (0.5 mg/kg/day in males, 5 mg/kg/day in females), or vehicle control (0.5% Tween in water).	0.5% Tween in water	Liver hepatosomatic index	Mice’s liver exposed to PFAS reported liver enlargement and a higher hepatosomatic index compared to the controls.
					Liver lipidomic	GenX-exposed group presented 199 lipids altered despite the low bioaccumulation in the liver. NBP2 affected 123 lipids. PFAS exposure leads to class- and sex-specific lipid changes: phosphatidylglycerols are elevated in most groups, while phosphatidylinositols are increased specifically in females. GenX enriches stress-related fatty acids as oleic acid and dihomo-γ-linoleic acid and other mono unsaturated fatty acids (MUFA). Consistently, GenX reduces acylcarnitines in males, indicating disrupted lipid signaling and mitochondrial β-oxidation.
[18]	Zebrafish larvae (Danio rerio)	PFHxS	One hour post-fertilization, Zebrafish embryos were exposed to PFHxS at concentrations of 0.01–10 μg/L and a solvent control (0.00001% DMSO) in E3 medium until 120 h post-fertilization.	E3 medium with 0.00001% DMSO (vehicle)	Relative liver size analysis	In PFHxS-exposed group relative liver size decreased significantly compared to the control group.
					Liver biochemical analysis	Increased levels of triglycerides (TG), total cholesterol (TC), and HDL-C were observed at 0.1 μg/L PFHxS. LDL-C levels increased significantly at 1 μg/L PFHxS
					Liver transcriptomic and gene expression	Transcriptomic analysis revealed several DEGs in the exposure group belonging to pathways associated with hepatic toxicity, steatosis, microvesicular hepatic steatosis, activation of hepatic stellate cells and remarkable liver damage. qRT-PCR highlighted profound dysregulation in the expression of genes involved in hepatic lipid metabolism, lipid oxidation, lipid transport and cholesterol metabolism.
[24]	Mice (C57BL/6; male)	PFOS	Mice were administered with PFOS in drinking water (3 μg/day) for 7 weeks and fed an isocaloric diet featuring inulin or pectin or cellulose	Drinking water + inulin; drinking water + pectin; drinking water + cellulose	Liver/Body Weight Ratio	Mice exposed to PFOS exhibited an increased liver/body weight ratio, independent of diet
					Liver lipidomic	PFOS-exposed groups reported an increase in hepatic levels of lysophosphatidylcholine, lysophosphatidylethanolamine, phosphatidylcholine, and ceramide (in control and pectin groups), while reducing sphingomyelin (SM) levels in control- and pectin-fed mice. Dietary fibers (inulin and pectin) generally mitigated PFOS-induced increases in lipids expression.
[26]	Mice (C57BL/6; male)	PFOA	Mice were divided into two groups and administered either with tap water (control group) or PFOA at a dose of 100 mg/kg body weight per day (treatment group) by oral gavage for three consecutive days.	Tap water	Liver lipidomic	PFOA-exposed group reported significant alterations in multiple hepatic lipid classes, with glycerophospholipids being the most affected. Among these, phosphatidylcholine, phosphatidylcholine, and triacylglycerol showed the greatest number of impacted species.
[22]	Mice (Ldlr−/−; male and female)	PFAS Mixture: PFOA, PFOS, PFHxS, PFNA, GenX	Male and female Ldlr mice were fed an atherogenic diet for 1 week prior to the beginning of PFAS exposure and then continued on the atherogenic diet for the remainder of the study. PFAS mixture exposure lasted for 7 weeks via their drinking water. Each of the five PFAS was present in the mixture water at a concentration of 2 mg/L and the mice were allowed to drink the water ad libitum.	Control water	Liver histopathology	PFAS-exposed groups (especially in the female mice) showed hypertrophy of the hepatocyte and lipid infiltration compared with vehicle control mice. In the 500 μg/L PFOS group accumulation of lipid droplets and increase in TAG levels were found. Both PFBS-treated groups induced similar global lipid changes in a dose-dependent manner, which were distinct from PFOS exposure; the result was an increase in phosphatidylcholines and sphingomyelins and a decrease in phosphatidylinositol.
					Body weight	PFAS-exposed females showed a marked reduction in body weight compared to control females
					Corporal composition	Fat weight was found to be significantly lower in PFAS-exposed mice (especially females) after 4 weeks and 7 weeks of PFAS exposure
					Liver weight	Liver weight was notably greater after 7 weeks of PFAS exposure in both groups
					Serum biochemical analysis	ALT plasma levels were significantly higher in PFAS-exposed females compared with controls
					Plasma biochemical analysis	Total cholesterol levels were significantly higher in female (415 mg/dL vs. 352 mg/dL) and male (488 mg/dL vs. 392 mg/dL) PFAS-exposed mice compared with control mice, respectively, after 7 weeks of PFAS exposure
						The HDL was notably higher in PFAS-exposed females (vehicle: 23mg/dL vs. PFAS-exposed group: 31 mg/dL), and in PFAS-exposed males (vehicle: 42 mg/dL vs. PFAS-exposed group: 50 mg/dL.
						There were remarkable positive correlations between PFOS and free VLDL/LDL and significant inverse correlations with HDL. Similar trends were observed for PFOA circulating levels
					Liver biochemical analysis	Hepatic levels of TC were significantly lower in PFAS-exposed groups compared with control mice after 7 wk of PFAS exposure
						Total hepatic bile acid levels showed a PFAS sex interaction: lower level in the female PFAS-exposed mice, whereas male PFAS-exposed mice were lower
					Liver and ileal protein expression	Hepatic levels of NTCP (Slc10a1) were significantly up-regulated in the PFAS-exposed males compared with male control groups
						Ileal levels of ASBT protein were significantly higher in males due to PFAS exposure
					PFAS quantification	The liver tissue had the highest content of PFBS compared to all examined tissues, reaching 0.9148 ± 0.1023 µg/g (ww); the RD-PFBS group showed a significant decrease in PFBS concentration compared to the PFBS group.
					Plasma and liver biochemical analysis	TG and TC levels in the liver were significantly lower in all test groups compared to the control group.
					Liver transcriptomic and gene expression	After 7 weeks of treatment, PFAS-exposed male and female mice showed altered expression of hepatic bile acid transporters (Abcc3, Abcc4, Slc10a1, Abcb11, and Ostβ) compared to the control groups. Ileum transcriptomic analysis reported 70 DEGs in PFAS-exposed mice related to the acute inflammatory response, fatty acid metabolic process e lipid metabolic process. Liver RT-PCR highlighted a significant reduction in FXR expression in PFAS-exposed mice compared to the controls. Other hepatic nuclear receptors (SHP, CAR e Gstm1) showed sex-dependent alterations.
[23]	Mice (C57BL/6; male)	PFBS	Mice were exposed to 50 μg/L PFBS via drinking water for 42 days (6 weeks). Animals were randomly assigned to four experimental groups: (1) water + normal diet (control), (2) water + normal diet + 50 μg/L PFBS (PFBS group), (3) water + 60% normal diet (RD group), and (4) water + 60% normal diet + 50 μg/L PFBS (RD-PFBS group)	Water	Liver weight	Relative liver weight increased significantly and in a dose-dependent manner in all groups exposed to PFOS and H-PFMO2OSA compared to the control group. The increase was notably greater in the H-PFMO2OSA groups than in the corresponding PFOS groups. Specifically, liver weight rose by 65.5% and 229.8% in the 1 and 5 mg/kg/day PFOS groups, and by 109% and 297.9% in the respective H-PFMO2OSA groups.
					PFAS quantification	Results showed that, although H-PFMO2OSA liver and serum concentrations were lower than those of PFOS, the relative liver weight in the H-PFMO2OSA groups was significantly higher than that in the corresponding PFOS groups.
[25]	Mice (C57BL/6; male)	PFOS or PFBS	Mice were exposed to 10 μg/L, 500 μg/L PFBS, or 500 μg/L PFOS for 28 days through drinking water	Pure water	Liver lipidomic	Lipidomic analysis revealed significant changes in 138, 238, and 310 lipids in the groups exposed to 10 μg/L PFBS, 500 μg/L PFBS, and 500 μg/L PFOS, respectively. PFBS exposure increased significantly the levels of phosphatidylcholines and sphingomyelins, and decreased the levels of phosphatidylinositols. PFOS (500 μg/L dose) exposure resulted in a significant increase in triacylglycerol levels.
[28]	Mice (BALB/c; male)	H-PFMO2OSA or PFOS	Mice were exposed by oral gavage once daily for 28 days to H-PFMO_2_OSA or PFOS at 0, 0.2, 1, or 5 mg/kg/day (7 groups total)	Milli-Q water	Liver histopathology	PFOA reduced hepatic steatosis in AB-wt embryos, while promoting steatosis in spns1-wt (+/+), (+/−) embryos, likely through impaired autophagy and elevated protein ubiquitination, leading to increased cellular stress
						HFBA exposure showed increased hepatic steatosis in AB-wt embryos compared with vehicle-treated embryos
					Liver and serum biochemical analysis	At the concentration of 100 μg/L, F-53B induced a significant increase in the levels of glucose, pyruvic acid, Cholesterol-Low Density Lipoproteins by 105.25%, triglycerides by 100% and total cholesterol by 56.98% in the liver compared to the control
[27]	Zebrafish (Wild type, AB strain, spns1-wt (+/+), spns1(+/−), spns1(−/−))	PFOA, HFBA and PTFA	Zebrafish embryos and spns1 mutant zebrafish embryo siblings spns1-wt (+/+), (+/−) and spns1 homozygous mutant spns1-mutant (−/−), 24 h post-fertilization (hpf) (n= 10–16), were exposed to PFAS (50–150 nM) such as PFOA, HFBA and PFTA for 48 h as indicated. PFOA or HFBA was directly added into the egg water (50 nM and 100 nM) or a combination containing PFOA, HFBA, and PFTA (50 nM each).	DMSO or Ethanol at maximum levels of treatments (*v*/*v*)	Liver histopathology	Livers of zebrafish exposed to 100 μg/L F-53B showed increased hepatic vacuolization, vacuolation density and deposit of lipid droplets compared to the control, index of steathosis
					Liver weight	The dose of 300 µg/kg body weight/day PFOA resulted in an increase in absolute and relative liver weight in male and female experimental models compared to the control
					Zebrafish embryos gene expression	PFOA exposure actively induces lipogenic genes as srebp1, pparg, lpin1a, fasn and scd1 in a dose-dependent manner with greater impact in spns1-mutant (−/−) zebrafish embryos compared to the other groups. HFBA exposure did not determine an increase expression of genes involved in lipid or lipogenic metabolism compared to the controls. The combined exposure of PFAS did not lead to significant changes in lipogenic gene expression in spns1-wt (+/+) and (+/−) embryos. In contrast, in spns1−/− embryos, the combined PFAS administration led to a significant downregulation of genes srebp1, fasn and scd1 in an autophagy-disregulated environment compared to the controls.
[30]	Zebrafish (Wild type; AB strain)	P-53B	Adult zebrafish were exposed to 0.25, 5, and 100 μg/L F-53B for 28 days	0.05% DMSO in water	Plasma and liver biochemical analysis	Female mice: reduction in plasma total cholesterol and triglycerides at 300 µg/kg body weight/day PFOA. Male mice: increase in hepatic tryglicerides at 300 µg/kg body weight/day PFOA and decrease in plasma triglycerides at either doses
					Liver histopathology	Confirmation of the quantitative increase in triglycerides in the liver of male mice administred with 300 µg/kg body weight/day PFOA
[31]	Mice (C57BL/6J; male and female)	PFOA	Mice were orally administereddminisatred 50 and 300 µg/kg body weight/day PFOA via drinking water for 28 days	Water	Liver and body weight	Low dose PFOA: no significant body weight gain. High dose PFOA: significant inhibition of weight gain from day 14 to 28. Low and high dose PFBA: no significant effects on body weight gain. Low and high dose PFOA: significant increase in liver weight with an increase in indices. Low dose PFBA: no significant effects on hepatic weight. High dose PFBA: significant increase in liver index
					PFAS quantification	The accumulation of PFOA and PFBA in mouse liver tissues increased proportionally with the administered dose. At the same exposure doses, PFOA tends to accumulate in greater quantity than PFBA.
[32]	Mice (C57BL/6J; male)	PFOA or PFBA	Mice were daily gavaged with 0.4 mg/kg body weight (low dose) or 10 mg/kg body weight (high dose) of PFOA or PFBA for 28 days	N.D.	Liver histopathology	In rats exposed to 100 and 1000 μg/L F–53 B: swelled cells and mild hepatic steatosis. In groups exposed to 10,100 and 1000: significantly higher deposition of lipid accumulation in hepatocytes compared to the control.
					Serum biochemical analysis	In rats exposed to 10, 100 and 1000 μg/L F–53 B: dose-dependent increase in ALT and AST, significant downregulation of lipoprotein ApoA and upregulation of lipoprotein ApoB compared to the control. In the 100 and 1000 μg/L F–53 B groups: significantly elevated levels of free fatty acids compared to the control. In all rats exposed to F-53B: significant increase in the levels of liver functional biomarkers (total bilirubin, cholinesterase and transforming growth factor-β1) compared to the control.
[35]	Rats (Sprague-Dawley; male and female)	F–53 B	F–53 B was prepared as a 2.5 mg/mL stock solution in DMSO and diluted with deionized water to create a gradient of concentrations (1, 10, 100, and 1000 μg/L). Each treatment group was administered with each concentration of F-53B via drinking water for 6 months.	0.004% DMSO in deionized water	Body and liver weight	No significant changes
					Liver histopathology	In rats exposed to 100 and 1000 μg/L F–53 B: swelled cells and mild hepatic steatosis. In groups exposed to 10, 100 and 1000: significantly higher deposition of lipid accumulation in hepatocytes compared to the control.
					Serum biochemical analysis	In rats exposed to 10, 100 and 1000 μg/L F–53 B: dose-dependent increase in ALT and AST, significant downregulation of lipoprotein ApoA and upregulation of lipoprotein ApoB compared to the control. In the 100 and 1000 μg/L F–53 B groups: significantly elevated levels of free fatty acids compared to the control. In all rats exposed to F-53B: significant increase in the levels of liver functional biomarkers (total bilirubin, cholinesterase and transforming growth factor-β1) compared to the control.
					Liver biochemical analysis	In rats exposed to 10, 100, and 1000 μg/L F–53 B: significantly higher levels of triglycerides and LDL-C compared to the control.
					Liver protein expression	In the 10, 100 and 1000 μg/L F-53B groups: enhanced expression of proteins involved in lipid synthesis (SREBP-1c, FASN, ACC) and lipolysis (ACOX1)
					Liver gene expression	At 10, 100 and 1000 μg/L F-53B: higher expression of genes involved in lipid metabolism (Fatty Acid Synthase and Acyl-CoA Oxidase1).
[37]	Mice (C57BL/6; male)	PFHxS	Mice were fed a high-fat diet and exposed to PFHxS via drinking water at doses ranging from 60 to 110 μg/kg of body weight for 12 weeks	Milli-Q water	Body weight and fat deposition	In the PFHxS-exposed group: significantly greater body weight gain from week 7th to 11th and deposition of subcutaneous fat compared to the control.
					Serum biochemical analysis	In the PFHxS-exposed group, ALT and LDL-C levels and the HOMA-IR index were significantly higher compared to the control group.
					Liver biochemical analysis	In the PFHxS-exposed group: significantly greater content of tryglycerides compared to the control
					Liver histopathology	In the PFHxS-exposed group: hepatocytes showed more distinct characteristics of degeneration, more consistent lipid deposition and steatosis.
[38]	Rats (Sprague-Dawley; male)	6:2 Cl-PFESA	Mice were administered 50 μg/kg body weight/day 6:2 Cl-PFESA for 28 days via intragastric infusion	Milli-Q water	Serum biochemical analysis	In the 6:2 Cl-PFESA-exposed group: significant reduction in the levels of AST and total serum proteins compared to the control
					Blood biochemical analysis	In the 6:2 Cl-PFESA-exposed group: significant increase in the red cell volume distribution width standard deviation, red cell volume distribution width coefficient of variation, neutrophil count and neutrophil percentage compared to the control.
[39]	Rats (Sprague-Dawley; male and female)	HFPO-TeA	Each group was administered with 5mL/kg bodyweight of HFPO-TeA at one of the eight half-log dose levels via oral gavage (0.3, 0.9, 2.3, 6.3, 17, 45.9, 124, and 335.2 mg/kg bodyweight) for 5 days	Water	Body and liver weight	Body weight loss was observed in the female group treated with 6.3 mg/kg/day and in both sexes in the 17 mg/kg group. Relative male liver weight increased at doses ranging from 0.9–6.3 mg/kg/day and decreased at 17 mg/kg/day compared to the control. Relative female liver weight increased with doses ≥ 0.3 mg/kg/day compared to the control.
					Clinical observation	In the male HFPO-Tea-exposed groups at doses 17 and 45.9 mg/kg/day: lethargy, piloerection, thinness, hunching, coldness to touch, abnormal breathing, and decreased movement. In the female HFPO-Tea-exposed group within the 6.3 mg/kg/day dose: piloerection and thinness. In the 17 mg/kg/day dose group: coldness to touch and hunching. In the 45.9 mg/kg/day: abnormal breathing. In the 124 mg/kg/day group: decreased movement and lethargy. In both sexes groups exposed to doses ≥ 45.9 mg/kg/day HFPO-Tea: premature death.
[40]	Mice (CD-1; male)	LiTFSI; PFOA	Mice were exposed for 14 days to 10 or 20 mg/kg body weight of LiTFSI or PFOA or for 30 days to 1 or 5 mg/kg body of LiTFSI and PFOA.	Corn oil	Liver weight	PFOA-exposed groups: relative liver weight increased dose-dependently in the 14 and 30 days exposure groups in concomitance with a dose-dependent body weight decreases compared to the control. LiTFSI-exposed groups: no significant changes in the 14 and 30 days groups compared to the control.
					Serum biochemical analysis	PFOA-exposed groups: at 10 mg/kg and 20 mg/kg for 14 days the ALT activity was, respectively, three and five times higher compared to the control. ALT activity was six times higher in the 5 mg/kg for 30 days. In the 14 days at 10 and 20 mg/kg dose groups, total serum bilirubin levels increased compared to the control. In both the 14 and 30 days groups: significant increase in the ratio of albumin/globin compared to the control. Glucose levels significantly decreased in both the 14 and 30 days groups. Cholesterol levels decrease in the 14-day 10 mg/kg group and in the 30-day exposure group. LiTSI-exposed groups: no significant changes expept for the increase in glucose and cholesterol levels in the 20mg/kg 14-day group.
					Liver gene expression	30 days exposure of PFOA and LiTFSI determined an increase in PPARα mRNA expression. PFOA treatment determined a significant increase in mRNA levels of Acot1, Acox1, and Acsl1.
[41]	Mice (C57BL/6; male and female)	PFOS	Male mice were fed ad libitum or in a 25% caloric deficit and administered with 100 µg/kg PFOS daily for 5 weeks	Tap water	Body and liver weight	PFOS treatment did not induce significant changes in the ad libitum or caloric deficit group compared to the respective control groups.
					Serum biochemical analysis	The mice group fed ad libitum and administered with PFOS reported a significant increase in adiponectin levels compared to its control group.
					Glucose tolerance test and insulin tolerance test	The group in a caloric deficit and treated with PFOS had significantly higher glucose levels within two hours compared to its control. Mice fed ad libitum or in a caloric deficit treated with PFOS showed an increase glucose load after 2.5 h compared to their respective controls.
					Liver gene expression	mRNA expressions of Irs1 e Glut2 were significantly lower in both PFOS-treated groups compared to their controls.
[29]	Mice (CD1; male and female)	HFPO-DA	Mice were treated with HFDO-DA 0, 0.1, 0.5, and 5 mg/kg for 84–85 days (males) and 53–65 days (females), in deionized water via gavage once daily.	Deionized water via oral gavage	Liver transcriptomic	Activation of PPARα signaling pathways, increased fatty acid metabolism (mitochondrial and peroxisomal β-oxidation) at low doses, downregulation of complement cascades, and altered cell cycle/apoptosis pathways at higher doses.
[16]	Rats (Sprague-Dawley; male and female)	2,3-Benzofluorene 6:1 fluorotelomer alcohol (FTOH) 10:2 FTOH perfluorohexanesulfonamide (PFHxSAm)	Rats were administered one of four study chemicals or vehicle control by oral gavage for five consecutive days (days 0–4). 2,3-Benzofluorene, 6:1 FTOH, and PFHxSAm were dosed at 0.15, 0.50, 1.40, 4, 12, 37, 111, 333, or 1000 mg/kg, while 10:2 FTOH was administered at 0.07, 0.20, 0.70, 2, 6, 18, 55, 160, or 475 mg/kg. Rats were euthanized on day 5 in random order.	Corn oil for 2,3-Benzofluorene and 6:1 FTOH and acetone:corn oil (1:99) for PFHxSAm and 10:2 FTOH	Liver transcriptomic and gene expression	PPAR-α target genes exhibited the highest dysregulation, with a predominant upregulation pattern. PFAS exposure altered lipid metabolism, inducing upregulation of fatty acid oxidation genes (Acadm, Acox1, Cpt2, Cyp4a1-3) and downregulation of lipid transport genes (Apoa1, Apoa5, Pltp). Male rats showed a sex-specific downregulation of the rate-limiting genes for gluconeogenesis (Pck1) and bile acid synthesis (Cyp7a1), while lipid synthesis (Scd) was upregulated in response to PFAS exposure.

## Data Availability

No new data were created or analyzed in this study. Data sharing is not applicable to this article.

## Abbreviations

The following abbreviations are used in this manuscript:

ABCB11	ATP-binding cassette, sub-family B member 11
ABCC3	ATP Binding Cassette Subfamily C Member 3
ABCC4	ATP Binding Cassette Subfamily C Member 4
ACADM	Acyl-Coenzyme A dehydrogenase
ACC	Acetyl-CoA carboxylase
ACLY	ATP Citrate Lyase
ACOX1	Acyl-CoA Oxidase 1
ALP	Alkaline phosphatase
ALT	Alanine transaminase
AMPK	AMP activated protein kinase
APOA1	Apolipoprotein A1
APOA5	Apolipoprotein A5
ASBT	Apical sodium-dependent bile acid transporter
AST	Aspartate aminotransferase
BAXA	BCL2 associated X, apoptosis regulator a
BSA	Bovine serum albumin
CAR	Chimeric antigen receptor
Cd14	Cluster of differentiation 14
Cd36	Cluster of differentiation 36
6:2 Cl-PFESA	6:2 Chlorinated perfluoroalkyl ether sulfonic acid
Col1α	Collagen type I alpha
CPT1A	Carnitine palmitoyltransferase 1A
CPT2	Carnitine palmitoyltransferase 2
Csf1ra	Colony stimulating factor 1 receptor, a
Cyp4a14	Cytochrome P450 omega-hydroxylase 4A14
Cyp4a1-3	Cytochrome P450 omega-hydroxylase 4A1-3
Cyp7a1	Cytochrome P450 omega-hydroxylase 7A1
DDIT3	DNA-damage-inducible transcript 3
DEGs	Differentially Expressed Genes
EDEM1	ER degradation enhancing alpha-mannosidase like protein 1
Ehhadh	Enoyl-CoA, Hydratase/3-Hydroxyacyl CoA Dehydrogenase
F-53B	6:2 Cl-PFESA
FABP1	Fatty Acid Binding Protein 1
FAT	Fatty Acid Transporter
FATP2	Fatty Acid Transport Protein 2
FASN	Fatty acid synthase
FGF-21	Fibroblast growth factor 21
FXR	Farnesoid X Receptor
GPAM	Glycerol-3-phosphate acyltransferase, mitochondrial
GGT	Gamma-glutamyl transferase
GOT	Glutamic-oxaloacetic transaminase
GPT	Glutamic-pyruvic transaminase
GSTM1	Glutathione S-transferase mu 1
HA	Humic acid
HDL	High density lipoprotein
HFBA	Heptafluorobutyric Acid
H-PFMO_2_OSA	Nafion byproduct 2
IL-1β	Interleukin-1 beta
IL-6	Interleukin-6
IL-8	Interleukin-8
LC3	Protein LC3
LDL	Low density lipoprotein
LiTFSI	Lithium bis(trifluoromethyl sulfonyl)imide
Lpl	Lipoprotein lipase
LXR	Liver X receptor
m-TOR	Mechanistic target of rapamycin
MAFLD	Metabolic dysfunction-Associated Fatty Liver Disease
MDA	Malondialdehyde
MCP-1	Monocyte chemoattractant protein-1
MTTP	Microsomal triglyceride transfer protein
MUFA	Mono unsatureated fatty acids
NEFA	Non-esterified fatty acids
Nfkbiaa	Nuclear factor of kappa light polypeptide gene enhancer in B-cells inhibitor, alpha a
NLRP3	NLR family pyrin domain containing 3
NPs	Nanoplastics
NTCP	Sodium taurocholate co-transporting polypeptide
OBS	Sodium p-perfluorous nonenoxybenzenesulfonate
OECD	Organisation for Economic Co-operation and Development
Ostb	Organic solute transporter beta
P62	Protein 62
PFAS	Per- and polyfluoroalkyl substances
PFBS	Perfluorobutanesulfonic acid
PFDA	Perfluorodecanoic acid
PFNA	Perfluorononanoic acid
PFHxS	Perfluorohexanesulfonic acid
PFOA	Perfluorooctanoic acid
PFOS	Perfluorooctanesulfonic acid
PFTrDA	Perfluorotridecanoic acid
PFuDA	Perfluoroundecanoic acid
PFTA	Perfluorotetradecanoic acid
PFTeDA	Perfluorotetradecanoic acid
PND	Post-natal day
PPAR	Peroxisome Proliferator-Activated Receptors
Prkcda	Protein kinase C delta type
PXR	Pregnane X receptor
RBP-4	Retinol-Binding Protein 4
RXR	Retinoic X receptor
ROS	Reactive Oxygen Species
SCD	Stearoyl-CoA desaturase
SLC10A1	Solute carrier family 10 member 1
SM	Sphingoyelin
Spns1	Spinster homolog 1
SREBP	Sterol Regulatory Element-Binding Protein
T-AOC	Total Antioxidant Capacity
TBA	Total bile acid
TC	Total cholesterol
TG	Triglycerides
TLR4	Toll-Like Receptor 4
TNF-α	Tumor Necrosis Factor-aplha
UPR	Unfolded protein response
VLDL	Very low density lipoprotein

## References

[1] Panieri E., Baralic K., Djukic-Cosic D., Djordjevic A.B., Saso L. (2022). PFAS Molecules: A Major Concern for the Human Health and the Environment. Toxics.

[2] OECD (2021). Reconciling Terminology of the Universe of Per- and Polyfluoroalkyl Substances.

[3] Corsini E., Iulini M., Galbiati V., Maddalon A., Pappalardo F., Russo G., Hoogenboom R.L.A.P., Beekmann K., Janssen A.W.F., Louisse J. (2024). EFSA Project on the Use of NAMs to Explore the Immunotoxicity of PFAS. EFSA Support. Publ..

[4] Nian M., Zhou W., Feng Y., Wang Y., Chen Q., Zhang J. (2022). Emerging and Legacy PFAS and Cytokine Homeostasis in Women of Childbearing Age. Sci. Rep..

[5] Fenton S.E., Ducatman A., Boobis A., DeWitt J.C., Lau C., Ng C., Smith J.S., Roberts S.M. (2021). Per- and Polyfluoroalkyl Substance Toxicity and Human Health Review: Current State of Knowledge and Strategies for Informing Future Research. Environ. Toxicol. Chem..

[6] Trefts E., Gannon M., Wasserman D.H. (2017). The Liver. Curr. Biol..

[7] Costello E., Rock S., Stratakis N., Eckel S.P., Walker D.I., Valvi D., Cserbik D., Jenkins T., Xanthakos S.A., Kohli R. (2022). Exposure to Per- and Polyfluoroalkyl Substances and Markers of Liver Injury: A Systematic Review and Meta-Analysis. Environ. Health Perspect..

[8] Das K.P., Wood C.R., Lin M.J., Starkov A.A., Lau C., Wallace K.B., Corton J.C., Abbott B.D. (2017). Perfluoroalkyl Acids-Induced Liver Steatosis: Effects on Genes Controlling Lipid Homeostasis. Toxicology.

[9] Schlezinger J.J., Puckett H., Oliver J., Nielsen G., Heiger-Bernays W., Webster T.F. (2020). Perfluorooctanoic Acid Activates Multiple Nuclear Receptor Pathways and Skews Expression of Genes Regulating Cholesterol Homeostasis in Liver of Humanized PPARα Mice Fed an American Diet. Toxicol. Appl. Pharmacol..

[10] Tricco A.C., Lillie E., Zarin W., O’Brien K.K., Colquhoun H., Levac D., Moher D., Peters M.D.J., Horsley T., Weeks L. (2018). PRISMA Extension for Scoping Reviews (PRISMA-ScR): Checklist and Explanation. Ann. Intern. Med..

[11] Wang Q., Huang J., Liu S., Wang C., Jin Y., Lai H., Tu W. (2022). Aberrant Hepatic Lipid Metabolism Associated with Gut Microbiota Dysbiosis Triggers Hepatotoxicity of Novel PFOS Alternatives in Adult Zebrafish. Environ. Int..

[12] Yang W., Ling X., He S., Cui H., Yang Z., An H., Wang L., Zou P., Chen Q., Liu J. (2023). PPARα/ACOX1 as a Novel Target for Hepatic Lipid Metabolism Disorders Induced by per- and Polyfluoroalkyl Substances: An Integrated Approach. Environ. Int..

[13] Khan E.A., Grønnestad R., Krøkje Å., Bartosov Z., Johanson S.M., Müller M.H.B., Arukwe A. (2023). Alteration of Hepato-Lipidomic Homeostasis in A/J Mice Fed an Environmentally Relevant PFAS Mixture. Environ. Int..

[14] Attema B., Janssen A.W.F., Rijkers D., van Schothorst E.M., Hooiveld G.J.E.J., Kersten S. (2022). Exposure to Low-Dose Perfluorooctanoic Acid Promotes Hepatic Steatosis and Disrupts the Hepatic Transcriptome in Mice. Mol. Metab..

[15] Lee W.K., Lam T.K.Y., Tang H.C., Ho T.C., Wan H.T., Wong C.K.C. (2023). PFOS-Elicited Metabolic Perturbation in Liver and Fatty Acid Metabolites in Testis of Adult Mice. Front. Endocrinol..

[16] Hari A., AbdulHameed M.D.M., Balik-Meisner M.R., Mav D., Phadke D.P., Scholl E.H., Shah R.R., Casey W., Auerbach S.S., Wallqvist A. (2024). Exposure to PFAS Chemicals Induces Sex-Dependent Alterations in Key Rate-Limiting Steps of Lipid Metabolism in Liver Steatosis. Front. Toxicol..

[17] Pan Z., Miao W., Wang C., Tu W., Jin C., Jin Y. (2021). 6:2 Cl-PFESA Has the Potential to Cause Liver Damage and Induce Lipid Metabolism Disorders in Female Mice through the Action of PPAR-γ. Environ. Pollut..

[18] Liao H., He Y.J., Zhang S., Kang X., Yang X., Xu B., Magnuson J.T., Wang S., Zheng C., Qiu W. (2024). Perfluorohexanesulfonic Acid (PFHxS) Induces Hepatotoxicity through the PPAR Signaling Pathway in Larval Zebrafish (Danio Rerio). Environ. Sci. Technol..

[19] Kirkwood-Donelson K.I., Chappel J., Tobin E., Dodds J.N., Reif D.M., DeWitt J.C., Baker E.S. (2024). Investigating Mouse Hepatic Lipidome Dysregulation Following Exposure to Emerging Per- and Polyfluoroalkyl Substances (PFAS). Chemosphere.

[20] Meng X., Yu G., Luo T., Zhang R., Zhang J., Liu Y. (2023). Transcriptomics Integrated with Metabolomics Reveals Perfluorobutane Sulfonate (PFBS) Exposure Effect during Pregnancy and Lactation on Lipid Metabolism in Rat Offspring. Chemosphere.

[21] He Y.J., Liao H., Yang G., Qiu W., Xuan R., Zheng G., Xu B., Yang X., Magnuson J.T., Schlenk D. (2024). Perfluorohexanesulfonic Acid (PFHxS) Impairs Lipid Homeostasis in Zebrafish Larvae through Activation of PPARα. Environ. Sci. Technol..

[22] Roth K., Yang Z., Agarwal M., Birbeck J., Westrick J., Lydic T., Gurdziel K., Petriello M.C. (2024). Exposure of Ldlr−/−Mice to a PFAS Mixture and Outcomes Related to Circulating Lipids, Bile Acid Excretion, and the Intestinal Transporter ASBT. Environ. Health Perspect..

[23] Liu S., Liu Y., Zhang D., Li H., Shao X., Xie P., Li J. (2023). Novel Insights into Perfluorinated Compound-Induced Hepatotoxicity: Chronic Dietary Restriction Exacerbates the Effects of PFBS on Hepatic Lipid Metabolism in Mice. Environ. Int..

[24] Deng P., Durham J., Liu J., Zhang X., Wang C., Li D., Gwag T., Ma M., Hennig B. (2022). Metabolomic, Lipidomic, Transcriptomic, and Metagenomic Analyses in Mice Exposed to PFOS and Fed Soluble and Insoluble Dietary Fibers. Environ. Health Perspect..

[25] Chen L., Liu Y., Mu H., Li H., Liu S., Zhu M., Bu Y., Wu B. (2022). Effects of Perfluorobutane Sulfonate and Perfluorooctane Sulfonate on Lipid Homeostasis in Mouse Liver. Environ. Pollut..

[26] Stoffels C.B.A., Angerer T.B., Robert H., Poupin N., Lakhal L., Frache G., Mercier-Bonin M., Audinot J.N. (2023). Lipidomic Profiling of PFOA-Exposed Mouse Liver by Multi-Modal Mass Spectrometry Analysis. Anal. Chem..

[27] Gadi S., Niture S., Hoang H., Qi Q., Hatcher C., Huang X., Haider J., Norford D.C., Leung T.C., Levine K.E. (2023). Deficiency of Spns1 Exacerbates Per- and Polyfluoroalkyl Substances Mediated Hepatic Toxicity and Steatosis in Zebrafish (Danio Rerio). Toxicology.

[28] Wang Z., Yao J., Guo H., Sheng N., Guo Y., Dai J. (2022). Comparative Hepatotoxicity of a Novel Perfluoroalkyl Ether Sulfonic Acid, Nafion Byproduct 2 (H-PFMO2OSA), and Legacy Perfluorooctane Sulfonate (PFOS) in Adult Male Mice. Environ. Sci. Technol..

[29] Heintz M.M., Chappell G.A., Thompson C.M., Haws L.C. (2022). Evaluation of Transcriptomic Responses in Livers of Mice Exposed to the Short-Chain PFAS Compound HFPO-DA. Front. Toxicol..

[30] Gu X., Yang H., Wu L., Fu Z., Zhou S., Zhang Z., Liu Y., Zhang M., Liu S., Lu W. (2024). Contribution of Gut Microbiota to Hepatic Steatosis Following F-53B Exposure from the Perspective of Glucose and Fatty Acid Metabolism. J. Hazard. Mater..

[31] Attema B., Kummu O., Pitkänen S., Weisell J., Vuorio T., Pennanen E., Vorimo M., Rysä J., Kersten S., Levonen A.L. (2024). Metabolic Effects of Nuclear Receptor Activation in Vivo after 28-Day Oral Exposure to Three Endocrine-Disrupting Chemicals. Arch. Toxicol..

[32] Wang X., Lv Y., Qiang X., Liang S., Li R., Zhan J., Liu J. (2024). Perfluorooctanoic Acid (PFOA) and Its Alternative Perfluorobutanoic Acid (PFBA) Alter Hepatic Bile Acid Profiles via Different Pathways. Sci. Total Environ..

[33] Maxwell D.A.L., Oluwayiose O.A., Houle E., Roth K., Nowak K., Sawant S., Paskavitz A.L., Liu W., Gurdziel K., Petriello M.C. (2024). Mixtures of Per- and Polyfluoroalkyl Substances (PFAS) Alter Sperm Methylation and Long-Term Reprogramming of Offspring Liver and Fat Transcriptome. Environ. Int..

[34] Blake B.E., Miller C.N., Nguyen H., Chappell V.A., Phan T.P., Phadke D.P., Balik-Meisner M.R., Mav D., Shah R.R., Fenton S.E. (2022). Transcriptional Pathways Linked to Fetal and Maternal Hepatic Dysfunction Caused by Gestational Exposure to Perfluorooctanoic Acid (PFOA) or Hexafluoropropylene Oxide-Dimer Acid (HFPO-DA or GenX) in CD-1 Mice. Ecotoxicol. Environ. Saf..

[35] Li X., Zhang Q., Wang A., Shan S., Wang X., Wang Y., Wan J., Ning P., Hong C., Tian H. (2024). Hepatotoxicity Induced in Rats by Chronic Exposure to F–53B, an Emerging Replacement of Perfluorooctane Sulfonate (PFOS). Environ. Pollut..

[36] Liu Y., Yu G., Medsker H., Luo T., Meng X., Wang C., Feng L., Zhang J. (2024). Perinatal Exposure to Perfluorooctane Sulfonate and the Risk of Hepatic Inflammation in Rat Offspring: Perturbation of Gut-Liver Crosstalk. Environ. Res..

[37] He X., Jiang J., Zhang X.X. (2022). Environmental Exposure to Low-Dose Perfluorohexanesulfonate Promotes Obesity and Non-Alcoholic Fatty Liver Disease in Mice Fed a High-Fat Diet. Environ. Sci. Pollut. Res..

[38] Zhao N., Kong Y., Yuan Q., Wei Z., Gu J., Ji C., Jin H., Zhao M. (2023). The Toxic Mechanism of 6:2 Cl-PFESA in Adolescent Male Rats: Endocrine Disorders and Liver Inflammation Regulated by the Gut Microbiota-Gut-Testis/Liver Axis. J. Hazard. Mater..

[39] Renyer A., Ravindra K., Wetmore B.A., Ford J.L., DeVito M., Hughes M.F., Wehmas L.C., MacMillan D.K. (2023). Dose Response, Dosimetric, and Metabolic Evaluations of Replacement PFAS Perfluoro-(2,5,8-Trimethyl-3,6,9-Trioxadodecanoic) Acid (HFPO-TeA). Toxics.

[40] Sands M., Zhang X., Laws M., Spinella M., Erdogan Z.M., Irudayaraj J. (2024). Comparative Hepatotoxicity of Novel Lithium Bis(Trifluoromethanesulfonyl)Imide (LiTFSI, Ie. HQ-115) and Legacy Perfluorooctanoic Acid (PFOA) in Male Mice: Insights into Epigenetic Mechanisms and Pathway-Specific Responses. Environ. Int..

[41] Salter D.M., Wei W., Nahar P.P., Marques E., Slitt A.L. (2021). Perfluorooctanesulfonic Acid (PFOS) Thwarts the Beneficial Effects of Calorie Restriction and Metformin. Toxicol. Sci..

[42] He X., Sun Z., Sun J., Chen Y., Luo Y., Wang Z., Linghu D., Song M., Cao C. (2024). Single-Cell Transcriptomics Reveal the Microenvironment Landscape of Perfluorooctane Sulfonate-Induced Liver Injury in Female Mice. Sci. Total Environ..

[43] Yi W., Shi J., Wang L., Wang D., Wang Y., Song J., Xin L., Jiang F. (2024). Maternal PFOS Exposure in Mice Induces Hepatic Lipid Accumulation and Inflammation in Adult Female Offspring: Involvement of Microbiome-Gut-Liver Axis and Autophagy. J. Hazard. Mater..

[44] Shao W., Xu J., Xu C., Weng Z., Liu Q., Zhang X., Liang J., Li W., Zhang Y., Jiang Z. (2021). Early-Life Perfluorooctanoic Acid Exposure Induces Obesity in Male Offspring and the Intervention Role of Chlorogenic Acid. Environ. Pollut..

[45] Roth K., Yang Z., Agarwal M., Liu W., Peng Z., Long Z., Birbeck J., Westrick J., Liu W., Petriello M.C. (2021). Exposure to a Mixture of Legacy, Alternative, and Replacement per- and Polyfluoroalkyl Substances (PFAS) Results in Sex-Dependent Modulation of Cholesterol Metabolism and Liver Injury. Environ. Int..

[46] Pfohl M., Marques E., Auclair A., Barlock B., Jamwal R., Goedken M., Akhlaghi F., Slitt A.L. (2021). An ‘Omics Approach to Unraveling the Paradoxical Effect of Diet on Perfluorooctanesulfonic Acid (PFOS) and Perfluorononanoic Acid (PFNA)-Induced Hepatic Steatosis. Toxicol. Sci..

[47] Jackson T.W., Lambright C.S., Evans N., Wehmas L.C., MacMillan D.K., Bangma J., Gray L.E., Conley J.M. (2024). Exploring Maternal and Developmental Toxicity of Perfluoroalkyl Ether Acids PFO4DA and PFO5DoA Using Hepatic Transcriptomics and Serum Metabolomics. Sci. Total Environ..

[48] Kaye E., Marques E., Agudelo Areiza J., Modaresi S.M.S., Slitt A. (2024). Exposure to a PFOA, PFOS and PFHxS Mixture during Gestation and Lactation Alters the Liver Proteome in Offspring of CD-1 Mice. Toxics.

[49] Junaid M., Liu S., Yue Q., Wang J. (2024). Exacerbated Interfacial Impacts of Nanoplastics and 6:2 Chlorinated Polyfluorinated Ether Sulfonate by Natural Organic Matter in Adult Zebrafish: Evidence through Histopathology, Gut Microbiota, and Transcriptomic Analysis. J. Hazard. Mater..

[50] United States Environmental Protection Agency (2021). United States Environmental Protection Agency Multi-Industry Per-and Polyfluoroalkyl Substances (PFAS) Study-2021 Preliminary Report.

[51] Zhang Y., Zhou Y., Dong R., Song N., Hong M., Li J., Yu J., Kong D. (2024). Emerging and Legacy Per- and Polyfluoroalkyl Substances (PFAS) in Fluorochemical Wastewater along Full-Scale Treatment Processes: Source, Fate, and Ecological Risk. J. Hazard. Mater..

[52] Thompson C.M., Fitch S.E., Ring C., Rish W., Cullen J.M., Haws L.C. (2019). Development of an Oral Reference Dose for the Perfluorinated Compound GenX. J. Appl. Toxicol..

[53] Li Y., Fletcher T., Mucs D., Scott K., Lindh C.H., Tallving P., Jakobsson K. (2017). Half-Lives of PFOS, PFHxS and PFOA after End of Exposure to Contaminated Drinking Water. Occup. Environ. Med..

